# Assessing the Diversity and Metabolic Potential of Psychrotolerant Arsenic-Metabolizing Microorganisms From a Subarctic Peatland Used for Treatment of Mining-Affected Waters by Culture-Dependent and -Independent Techniques

**DOI:** 10.3389/fmicb.2021.648412

**Published:** 2021-07-06

**Authors:** Aileen Ziegelhöfer, Katharina Kujala

**Affiliations:** ^1^Faculty for Chemistry & Biotechnology, Aachen University of Applied Sciences, Jülich, Germany; ^2^Water, Energy and Environmental Engineering Research Unit, University of Oulu, Oulu, Finland

**Keywords:** peatland, mining wastewater treatment, psychrotolerant microorganisms, arsenic metabolizing microbes, dilution to extinction, isolation

## Abstract

Arsenic contamination in water by natural causes or industrial activities is a major environmental concern, and treatment of contaminated waters is needed to protect water resources and minimize the risk for human health. In mining environments, treatment peatlands are used in the polishing phase of water treatment to remove arsenic (among other contaminants), and peat microorganisms play a crucial role in arsenic removal. The present study assessed culture-independent diversity obtained through metagenomic and metatranscriptomic sequencing and culture-dependent diversity obtained by isolating psychrotolerant arsenic-tolerant, arsenite-oxidizing, and arsenate-respiring microorganisms from a peatland treating mine effluent waters of a gold mine in Finnish Lapland using a dilution-to-extinction technique. Low diversity enrichments obtained after several transfers were dominated by the genera *Pseudomonas*, *Polaromonas*, *Aeromonas*, *Brevundimonas*, *Ancylobacter*, and *Rhodoferax*. Even though maximal growth and physiological activity (i.e., arsenite oxidation or arsenate reduction) were observed at temperatures between 20 and 28°C, most enrichments also showed substantial growth/activity at 2–5°C, indicating the successful enrichments of psychrotolerant microorganisms. After additional purification, eight arsenic-tolerant, five arsenite-oxidizing, and three arsenate-respiring strains were obtained in pure culture and identified as *Pseudomonas*, *Rhodococcus*, *Microbacterium*, and *Cadophora*. Some of the enriched and isolated genera are not known to metabolize arsenic, and valuable insights on arsenic turnover pathways may be gained by their further characterization. Comparison with phylogenetic and functional data from the metagenome indicated that the enriched and isolated strains did not belong to the most abundant genera, indicating that culture-dependent and -independent methods capture different fractions of the microbial community involved in arsenic turnover. Rare biosphere microorganisms that are present in low abundance often play an important role in ecosystem functioning, and the enriched/isolated strains might thus contribute substantially to arsenic turnover in the treatment peatland. Psychrotolerant pure cultures of arsenic-metabolizing microorganisms from peatlands are needed to close the knowledge gaps pertaining to microbial arsenic turnover in peatlands located in cold climate regions, and the isolates and enrichments obtained in this study are a good starting point to establish model systems. Improved understanding of their metabolism could moreover lead to their use in biotechnological applications intended for bioremediation of arsenic-contaminated waters.

## Introduction

Arsenic contamination of water bodies is a major environmental and health concern (Bissen and Frimmel, 2003). Some waters are naturally rich in arsenic, while others are contaminated through human activities. One such activity is metal mining. Often ores (e.g., gold-containing ores) contain arsenic which is released into the environment as a consequence of the ore-beneficiation process or through leaching from waste rock material (Bissen and Frimmel, 2003; Nordstrom, 2011). Removal of arsenic is necessary before mining-affected waters can be safely discharged into receiving water bodies. Along with active treatment which often relies on precipitation and/or absorption of arsenic, passive treatment in constructed wetlands has been used for arsenic removal from mining-affected waters. In Northern Finland, treatment wetlands constructed on formerly natural peatlands (treatment peatlands) are a common form of passive treatment (Palmer et al., 2015; Kujala et al., 2019). Arsenic removal in treatment peatlands relies on biological processes and microbial turnover of different arsenic species, as the sorption and binding of arsenic to peat material depend strongly on arsenic speciation (Langner et al., 2012; Besold et al., 2018). Microorganisms can use the arsenic oxoacids arsenite and arsenate in the energy-conserving process of arsenite oxidation and dissimilatory arsenate reduction (arsenate respiration), respectively (Oremland and Stolz, 2003; Stolz et al., 2006). The potential for microbial arsenite oxidation and arsenate reduction has been observed in peatlands (Kujala et al., 2020). In addition, arsenic-tolerant microbes are important drivers of many other contaminant removal processes like nitrogen or sulfate removal in treatment peatlands and other high-arsenic environments. They can, moreover, promote plant growth and phytoremediation in high-arsenic environments (Mesa et al., 2017; Upadhyay et al., 2018).

To date, only few studies have targeted the isolation of arsenic-metabolizing microbes from peatlands, and most arsenic-metabolizing pure cultures have been isolated from environments like arsenic-contaminated groundwaters, sediments, or hot springs (e.g., Salmassi et al., 2006; Pepi et al., 2007; Kaushik et al., 2012; Cordi et al., 2015). Isolates can be used as model organisms to study arsenic turnover processes in peat. They can moreover be used for bioaugmentation to improve the arsenic removal performance of peat-based water treatment systems. Thus, the current study aims to enrich and isolate arsenite-oxidizing, arsenate-respiring, and arsenic-tolerant microorganisms from peat soil using a dilution-to-extinction approach. In this approach, liquid media are inoculated with dilutions of soil or water samples sufficiently high to inoculate each incubation with one to a few cells only without pre-enrichment. There is thus a higher chance to isolate microorganisms that are highly abundant in peat that might be outcompeted by less abundant fast-growing microorganisms in classical enrichment approaches. The dilution-to-extinction approach has been successfully applied to isolate microorganisms from other environments such as marine and freshwater systems, soils, and sediments and has led to the isolation of formerly uncultured microorganisms (e.g., Connon and Giovannoni, 2002; Colin et al., 2013; Castro et al., 2017; Henson et al., 2018, 2020).

The main objectives of the current study were thus to (i) enrich and isolate arsenic-tolerant, arsenite-oxidizing, and arsenate-respiring microorganisms from peat soil using a dilution-to-extinction approach, (ii) identify the isolated microorganisms, (iii) assess the effect of temperature and arsenic concentration on their growth and activity, (iv) select promising isolates that might serve as model organisms in future studies for further characterization, and (v) compare the diversity of arsenic-metabolizing microorganisms based in this culture-dependent approach to the diversity based on culture-independent metagenome and metatranscriptome sequencing.

## Materials and Methods

### Origin of Peat Used for Cultivation and Molecular Analyses

Peat was obtained from a treatment peatland that has been used to purify pre-treated process wastewaters at a gold mine in Finish Lapland since 2010. Arsenic-metabolizing microbes and arsenic-turnover potentials have been demonstrated for peat from this treatment peatland in an earlier study (Kujala et al., 2020). Inflow waters to the peatland contain arsenic in concentrations ranging from 1 to 400 μg l^–1^. Arsenate is the major arsenic species in inflow waters (>95%). Moreover, inflow waters contain various other contaminants, including sulfate (2 g l^–1^), nitrate (7.2 mg l^–1^ nitrate-N), and ammonium (19 mg l^–1^ ammonium-N). Inflow waters are slightly basic (pH 7.8), which has led to elevated pH in the peat porewater in the treatment peatland (pH 6–7) compared to surrounding peatland areas not inundated with process wastewater (pH 45). A more detailed site description can be found in Palmer et al. (2015) and Khan et al. (2020). Samples were taken from the upper 10 cm of peat near the process water inflow point in July 2018 for metagenomic and metatranscriptomic analysis (triplicate soil cores) and in March 2020 for cultivation studies, as highest peat arsenic concentrations have been observed near the inflow (150–600 mg kg^–1^_*DW*_ in 2020; see also Palmer et al., 2015). Samples for metagenomic and metatranscriptomic analyses were frozen on site using dry ice and stored frozen until further processing. Samples for cultivation studies were stored cooled and processed within 2 weeks of sample collection.

### Dilution-to-Extinction Cultivation

Growth media were prepared using an artificial porewater solution containing essential minerals, trace elements, and vitamins (described in Kujala et al., 2020) supplemented with 10 mM arsenite, 10 mM arsenate, and 1:10 diluted nutrient broth (NB, Sigma-Aldrich; to a final concentration of 2.5 g l^–1^) for aerobic arsenic-tolerant microorganisms, with 2 mM arsenite and 2 mM HCO_3_^–^ as the sole carbon source for chemolithoautotrophic arsenite-oxidizing microorganisms and with 2 mM arsenate, 2 mM lactate, and 2 mM acetate for arsenate-respiring microorganisms. In the medium for arsenate-respiring microorganisms, the oxygen concentration was minimized by purging the medium with inert nitrogen gas during preparation. The pH of all growth media was adjusted to 6.5, which corresponds to the average pH measured *in situ* at the sampling site.

All isolation approaches were set up in 96-well plates. Four to five plates per target group were inoculated with different dilutions of a peat suspension (Table 1). The dilution steps used were based on most probable number counts obtained in an earlier study with peat soil from the same site, which were approximately 10^7^, 10^5^, and 10^6^ cells g_DW_^–1^ for arsenic-tolerant, arsenite-oxidizing, and arsenate-respiring microorganisms, respectively (Kujala et al., 2020). In each well, 270 μl of the respective growth medium was inoculated with 30 μl from the dilution series. On each plate, eight wells were inoculated with sterile water to serve as negative controls.

**TABLE 1:** Setup of the dilution-to-extinction cultivation approaches and their success rates.

Isolation approach	Dilution step	Additional pre-treatment	Approximate no. of culturable cells/well^*a*^	No. of wells inoculated	No. of positive wells
Arsenic-tolerating microorganisms (As-tol)	10^–2^	None	300	88	88 (100%)
	10^–2^	Filtered^b^	n.a.	88	2 (2%)
	10^–3^	None	30	88	88 (100%)
	10^–4^	None	3	176	99 (56%)

Arsenite-oxidizing microorganisms (As-ox)	10^–2^	None	3	88	30 (34%)
	10^–2^	Filtered^b^	n.a.	88	0 (0%)
	10^–3^	None	0.3	88	13 (15%)
	10^–4^	None	0.03	88	2 (2%)

Arsenate-respiring microorganisms (As-red)	10^–3^	None	3	88	58 (66%)
	10^–3^	Filtered^b^	n.a.	88	14 (16%)
	10^–4^	None	0.3	88	50 (57%)
	10^–5^	None	0.03	88	8 (9%)

*^*a*^Calculations based on MPN counts (Kujala et al., 2020) and an inoculation volume of 30 μl per well.*

*^*b*^Sample was passed through a 0.2-μm filter prior to inoculation.*

Isolation approaches for arsenite-oxidizing and arsenic-tolerant microorganisms were incubated in ambient air. Isolation approaches for arsenate-respiring microorganisms were incubated in air-tight containers in which an anoxic atmosphere was created by the use of AnaeroGen pouches (Thermo Fisher Scientific, Waltham, MA, United States). All approaches were incubated at 10°C, which represents the average summer *in situ* temperature in the upper peat layers. After approximately 1 month, 30 μl spent growth medium was transferred from each well into fresh medium. This transfer was done at least three times in total. After the last transfer, plates were incubated at 20°C to allow for faster growth of the isolates to obtain enough starting material for screening, physiological tests and PCR of 16S rRNA genes.

### Screening of Plates for Arsenic-Metabolizing Enrichments

Possible growth of the target groups in the 96-well plates was monitored by optical density (OD) measurements at 600 nm (all approaches) and tests for arsenite/arsenate turnover (arsenite-oxidizing/arsenate-respiring microorganisms). Wells from the approach targeting arsenic-tolerant microorganisms were scored positive based on growth observed by visual inspection and OD measurements. Arsenite-oxidizing and arsenate-respiring microorganisms were slow growing and did not reach high OD during the 4-week incubation period, but arsenite/arsenate turnover indicated their continued presence and activity after each transfer. Wells were scored as positive or negative for arsenite oxidation/arsenate respiration using a KMnO_4_ screening approach (Salmassi et al., 2002, 2006). Up to 40 μl of 0.1 M KMnO_4_ was added to 100 μl of spent growth medium in 2-μl increments. The presence of arsenate was indicated by purple and the presence of arsenite by orange/yellow color. Wells from the approach targeting arsenite-oxidizing microorganisms were scored positive, if the KMnO_4_ test indicated substantial amounts of arsenate in the growth medium, and wells from the approach targeting arsenate-respiring microorganisms were scored positive, if the KMnO_4_ test indicated substantial amounts of arsenite in the growth medium. From the approaches targeting arsenite-oxidizing and arsenate-respiring microorganisms, all wells showing arsenite oxidation or arsenate respiration activity, respectively, were chosen for further characterization, while from the approach targeting arsenic-tolerant microorganisms only growth-positive wells from the highest dilution and some wells of the second-to-highest dilution were chosen, as using the lower dilutions would likely have meant an increased probability of mixed cultures in the wells (Table 1).

### Isolation of Arsenic-Metabolizing Strains

Further isolation of arsenic-metabolizing strains from the enrichments was achieved by plating 100 μl from growth or activity positive wells onto agar plates prepared with media of the same composition as for the liquid cultivation. Plates were incubated at 20°C, and single colonies were transferred to fresh agar plates using the streak plating technique. Time between transfers ranged from <1 week to >2 months due to the difference in growth rates of the strains; strains were considered pure cultures after three transfers. To date, pure cultures were obtained for some of the faster-growing strains, while for most of the slow-growing strains further transfers are still needed to reach the pure culture status. Thus, only the faster-growing strains were further characterized as part of this study.

### Identification of Dominant Strains in Enrichments and Isolates

The dominant strains in the enrichments as well as the isolates obtained after streak plating were identified through Sanger sequencing of their 16S rRNA genes or the internal transcribed spacer (ITS) region of the rRNA genes. The spent growth medium containing microbial cells (enrichments) or colonies suspended in PCR-grade water (isolates) was directly used for PCR amplification with primers 27F-1492R or ITS1–ITS4 (White et al., 1990; Lane, 1991). Prior to amplification, cell suspensions were heated to 95°C and kept at this temperature for 10 min to improve amplification efficiencies in direct PCR. PCR products were sent to Macrogen Europe BV (Amsterdam, Netherlands) for purification and subsequent sequencing. Isolates were sequenced using both forward and reverse primers, while enrichments were sequenced using the forward primer only. Sequencing trace files were visually inspected, and low-quality sequences (i.e., low signal, strongly mixed signal, or <300 bp length) were removed prior to further analysis. Special attention was paid to identify sequences that had mixed base calls, as this potentially indicates the presence of more than one species in the enrichments. Enrichments with sequences that did not indicate the presence of more than one strains were marked as “potential pure culture” (Supplementary Tables 1–3). 16S rRNA gene sequences were trimmed at the 5′ and 3′ ends to remove noisy bases, resulting in an average final sequence length of 800 bp (range 500–1,100 bp). For isolates, forward and reverse reads were merged in MEGA 7 (Kumar et al., 2016) to obtain full-length sequences. The sequences were classified using the SILVA Alignment, Classification and Tree Service (Quast et al., 2013)^1^ or PROTAX-fungi (Abarenkov et al., 2018), and most closely related 16S rRNA gene reference sequences were identified using BLAST. Phylogenetic trees with 1,000 bootstrap replications were inferred from the isolates’ and reference sequences in MEGA 7 (Kumar et al., 2016) using the neighbor-joining method and evolutionary distances calculated using the maximum composite likelihood algorithm (Saitou and Nei, 1987; Tamura et al., 2004). Genus-level diversity measures (Shannon diversity and Pielou’s evenness) were calculated using only the isolates for which sequencing had been successful. The 16S rRNA gene and ITS sequences obtained in this study were deposited in the National Center for Biotechnology Information (NCBI) under accession numbers MW496900–MW497062 (enrichments), MW800181–MW800195 (bacterial isolates), and MW800602 (fungal isolate).

### Temperature Ranges for Growth and Activity of Eenriched Microorganisms

Selected enrichments were inoculated in the respective medium and incubated at different temperatures to test for their growth (arsenic-tolerant microorganisms) or activity (arsenite-oxidizing/arsenate-respiring microorganisms) range. Incubation temperatures varied from 2 to 40°C. Growth rates of arsenic-tolerant microorganisms were determined by OD measurements, while arsenite/arsenate turnover rates were determined by measurement of arsenate and arsenite concentrations in the growth medium over the course of several days to weeks. Arsenate concentrations as well as total arsenic concentrations were determined using a colorimetric assay (Dhar et al., 2004). The measured concentration of total arsenic was stable over time, while arsenate concentrations increased or decreased over time. For arsenite-oxidizing microorganisms, an increase in arsenate concentration indicated arsenite oxidation, while for arsenate-respiring microorganisms a decrease in arsenate concentration indicated arsenate respiration.

A semiquantitative comparison of growth rates, arsenite oxidation, and arsenate reduction was attempted to allow for visualization of the preferred temperature range of the enrichments and to identify enrichment isolates with the fastest arsenic turnover or growth. For this comparison, all observed growth and turnover rates were divided by the highest growth/turnover rate observed for the respective isolation approach and scored as follows: (−) no observed growth/activity (0–2% of maximum), (+) low growth/activity (2%–24% of maximum), (++) substantial growth/activity (25–49% of maximum), (+++) high growth/activity (50–74% of maximum), and (++++) maximum growth/activity (75–100% of maximum).

### Assessment of the Isolates’ Tolerance to Arsenite and Arsenate

Isolates from the approach targeting arsenic-tolerant microorganisms were tested for their tolerance to high levels of arsenite or arsenate in the medium. Tests were conducted in 96-well plates containing 250 μl of the same medium that had been used for isolation. To this medium, arsenite or arsenate was added to reach final concentrations ranging from 10 to 200 mM arsenite/arsenate. Strains were pre-grown in basic medium for arsenic-tolerant microorganisms to OD of 0.3–0.4 after which 20 μl was used to inoculate the wells of the 96-well plates. OD was measured in regular intervals.

### Assessment of Arsenite Oxidation and Arsenate Reduction Potentials of the Isolated Strains

Arsenite oxidation potential of the isolates was tested in oxic incubations prepared with artificial porewater, 2 mM HCO_3_^–^, and arsenite concentrations ranging from 45 to 330 μM. Incubations were conducted in 24-well plates. Wells were inoculated with cell material pre-grown on agar plates and suspended in artificial porewater, incubated at 20°C in the dark on an orbital shaker, and sampled at regular intervals. Arsenate and total arsenic concentrations were determined using a colorimetric assay (Dhar et al., 2004). Rates were calculated based on arsenate production in the incubation tubes and normalized against the OD of the first sampling timepoint to account for possible differences in rates due to slight variation of microbial cell concentrations in the inoculum. Since OD for the isolates were not calibrated against cell counts and OD measurements likely correspond to different cell numbers for the different isolates, this normalization was used mainly to compare incubations with different arsenite concentrations of the same isolate.

Arsenate reduction potential of the isolates was tested in anoxic incubations prepared with artificial porewater, 2 mM lactate, and acetate and arsenate concentrations ranging from 45 to 330 μM. In addition, an artificial mine water medium was prepared with the following composition: 2 g l^–1^ NaSO_4_, 25 mg l^–1^ NH_4_Cl, 60 mg l^–1^ NaNO_3_, 50 μM arsenate, 1 mM acetate, 1 mM lactate, pH 7.0. The oxygen concentration was minimized by purging the medium with inert nitrogen gas during preparation. Incubations were prepared in balch-type incubation tubes sealed with gas-tight rubber stoppers with nitrogen gas in the headspace. Tubes were inoculated with cell material suspended in artificial porewater, incubated at 20°C in the dark on an orbital shaker, and sampled in regular intervals. Arsenate and total arsenic concentrations were determined using a colorimetric assay (Dhar et al., 2004), and rates were calculated based on arsenate concentrations and normalized as described above.

### Metagenomic and Metatranscriptomic Analysis

Total DNA and RNA were extracted from triplicate soil samples using the MoBio RNA PowerSoil Total RNA Isolation Kit and the RNA PowerSoil DNA Elution accessory Kit according to the manufacturer’s instructions. Bacterial ribosomal RNA was removed from the extracted RNA using the Ribo-Zero rRNA removal kit for Bacteria (Illumina, San Diego, CA, United States), and cDNA was generated from rRNA-depleted RNA using the SuperScript IV VILO MasterMix (Thermo Fisher Scientific). Triplicate DNA extracts and cDNA preparations were pooled and sent to Macrogen Europe B.V. (Amsterdam, Netherlands) for shotgun metagenome and metatranscriptome sequencing. A 150-bp paired-end sequencing was performed on Illumina HiSeq X, yielding approximately 23 and 4 Gb of raw sequence reads in the metagenomic and metatranscriptomic libraries, respectively. Forward and reverse read FastQ files were uploaded to the Metagenomic Rapid Annotations using the Subsystems Technology (MG-Rast) server for sequence quality trimming and analysis (Meyer et al., 2008). In MG-Rast, taxonomic profiling of the processed reads was done by annotating all reads or 16S rRNA reads with the RefSeq or SILVA SSU database, respectively (Quast et al., 2013; O’Leary et al., 2016), using default analysis parameters. Functional profiling was done by annotating all reads with the KEGG Orthology database in MG-Rast using default parameters. Quality-trimmed sequence data were downloaded from MG-Rast and searched against a custom database generated from 16S rRNA gene sequences of the enrichments and isolates as well as custom databases containing sequences of the functional genes *arsC*, *aioA*, or *arrA* from references downloaded from repositories and sequences obtained in a previous study (Kujala et al., 2020) using BLAST+ (Camacho et al., 2009). Parameters for the 16S rRNA gene and functional search were as follows: “blastn” (16S)/”blastx” (functional genes) with default parameters except for “pident” = 100 (16S; only allowing 100% matches)/80 (functional genes) and “max_target_seqs” = 200 (16S)/1 (functional genes). Detected functional genes were limited to those with a read length ≥100 bp. The metagenome and metatranscriptome sequence data are available at MG-Rast under project numbers mgm4838788.3, mgm4928889.3, and mgm4928890.3.

## Results and Discussion

### Microbial Community Composition and Metabolic Potential in Peat

**FIGURE 1:**
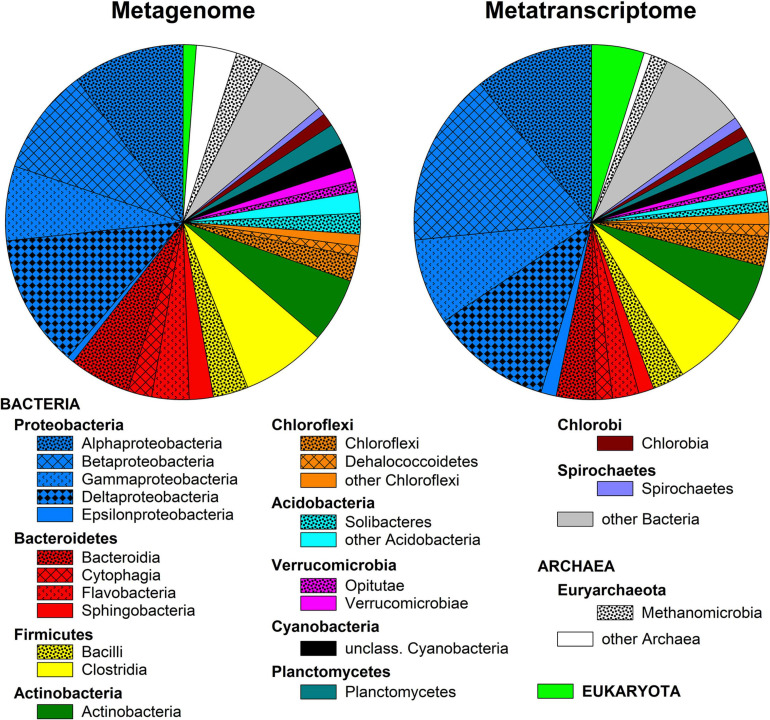
Class-level diversity of the microbial community in surface layer peat soil based on annotation of metagenomic and metatranscriptomic reads against the RefSeq database in MG-Rast. Only classes with a relative abundance >1% are presented.

Metagenomic and metatranscriptomic sequencing generated approximately 150,000,000 and 45,000 sequences, respectively, with an average sequence length of 155 bp. Eighty-five percent of the metagenomic but only 40% of the metatranscriptomic sequences passed quality control in MG-Rast and were used for downstream analyses. The majority of the sequence reads in both the metagenome and metatranscriptome were annotated to Bacteria (92.5 and 91.1% in metagenome and metatranscriptome, respectively) against the RefSeq database, while Archaea (6.2 and 4.2%, respectively) and Eukaryota (1.2 and 4.8%, respectively) were less abundant. The microbial community was dominated by Proteobacteria (40 and 47%, respectively), Bacteroidetes (14 and 9.1%, respectively), Firmicutes (12 and 10.1%, respectively), Actinobacteria (6 and 7.2%, respectively), and Euryarchaeota (5 and 3.4%, respectively; Figure 1). Alpha-, Beta-, Gamma-, and Delta-Proteobacteria were of similar relative abundance in the metagenome (7–12%), while in the metatranscriptome Beta-Proteobacteria had a slightly higher relative abundance (16% vs. 8–11%). Within the Bacteroidetes, Bacteroidia, Cytophagia, Flavobacteria, and Sphingobacteria were detected both in the metagenome and in the metatranscriptome (Figure 1).

Metagenomic and metatranscriptomic reads were annotated to 1,273 and 1,466 genera, respectively, of which only 10 and 9, respectively, had a relative abundance greater 1%. Genus-level diversity was high (Shannon index 5.78 and 5.89 for metagenome and metatranscriptome, respectively; Table 2), and the community had a rather even distribution (evenness 0.81 for both metagenome and metatranscriptome). The three most abundant genera in the metagenome were *Bacteroides* (2.9%), *Geobacter* (2.6%), and Candidatus *Solibacter* (1.9%), while the three most abundant genera in the metatranscriptome were *Thiobacillus* (2.6%), *Bacteroides* (1.9%), and *Acidovorax* (1.8%). *Geobacter* spp. are known for detoxifying and dissimilatory arsenate reduction (Dang et al., 2017; Tsuchiya et al., 2019), and *Geobacter*-related dissimilatory arsenate reductase genes have been detected in the treatment peatland (Kujala et al., 2020).

**TABLE 2:** Genus-level diversity of metagenomic and metatranscriptomic peat microbial communities, enrichments, and functional genes involved in arsenic metabolism expressed as Shannon diversity and Pielou’s evenness.

		Shannon diversity index	Pielou’s evenness
Peat microbial community	Metagenome	5.78	0.81
	Metatranscriptome	5.89	0.81
Enrichments	Arsenic-tolerant microorganisms	1.70	0.37
	Arsenite-oxidizing microorganisms	1.64	0.53
	Arsenate-respiring microorganisms	0.79	0.20
Functional genes	*arsC*	3.92	0.73
	*aioA*	3.80	0.80
	*arrA*	1.30	0.38

*For enrichments, diversity measures were calculated based on those enrichments for which sequence information was available.*

**FIGURE 2:**
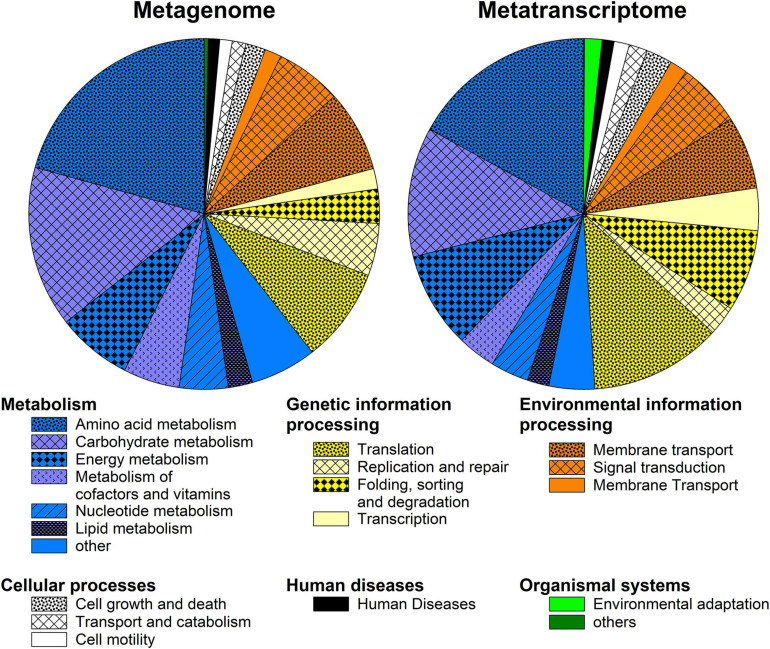
Functional profiles in the peat metagenome and metatranscriptome based on KEGG Orthology annotations in MG-Rast. The most abundant level 2 pathways are displayed.

The metagenome and metatranscriptome were annotated using the KEGG orthology database in MG-Rast. Functional profiles for the metagenome and metatranscriptome were rather similar, with most reads annotated to the KEGG categories of metabolism (60 and 50%, respectively; mainly amino acid metabolism, carbohydrate metabolism, and energy metabolism), genetic information processing (19 and 26%, respectively), environmental information processing (15 and 14%, respectively), and cellular processes (4 and 6%, respectively; Figure 2). Pathways or enzymes related to arsenic metabolism were not found in the KEGG-annotated metagenome or metatranscriptome, likely due to a rather low prevalence of these pathways.

### Enrichment, Isolation, and Identification of Arsenic-Metabolizing Microorganisms From Peat

Based on observed growth, arsenite oxidation activity, or arsenate reduction activity, 117, 40, and 76 low-diversity enrichments were obtained from the approaches targeting arsenic-tolerant, arsenite-oxidizing, and arsenate-respiring microorganisms, respectively, after at least three transfers. Arsenic-tolerant microorganisms showed much faster growth based on OD than the other two groups which did not grow to high OD within the given 4-week time frame between transfers. Good-quality sequencing results of 16S rRNA genes were obtained for 100 arsenic-tolerant, 22 arsenite-oxidizing, and 51 arsenate-respiring enrichments (Supplementary Tables 1–3). Many of the 16S rRNA sequences did not show signs of mixed cultures, indicating that the microbial communities in the enrichments were of very low diversity, each strongly dominated by one strain (Supplementary Tables 1–3). Enrichments from all approaches mainly belonged to the *Alpha*- and *Gamma-Proteobacteria*, while only a few belonged to the *Actinobacteria* (2% of all isolates with successful sequencing). About half of the enrichments were considered to be potential pure cultures based on the obtained sequences. The sequence identity with the most closely related pure cultures ranged from 79 to 99%. In some cases, low sequence identity might be due to the presence of a mixed culture which produced a mixed 16S rRNA gene sequence. However, some potential pure cultures also showed sequence identities of only around 90% with the next-related reference sequences. From the enrichments, eight arsenic-tolerant, five arsenite-oxidizing, and three arsenate-respiring pure culture strains were obtained. All of these isolates were fast growing, allowing for transfers in intervals of approximately 1 week. Isolation of slower-growing arsenic-metabolizing microorganisms from the enrichment cultures is still ongoing.

**FIGURE 3:**
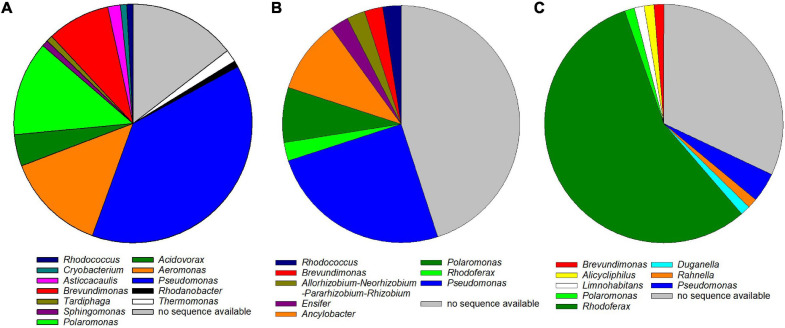
Genus-level classification of arsenic-tolerant **(A)**, arsenite-oxidizing **(B)**, and arsenate-respiring **(C)** microorganisms in the enrichments. The 16S rRNA genes of dominant strains in 117, 45, and 76 enrichments, respectively, were sequenced and classified using the SILVA Alignment, Classification, and Tree service. For some enrichments, no sequence was available because PCR amplification of the 16S rRNA gene was not successful or because the obtained sequence was of low quality.

Arsenic-tolerant microorganisms showed the highest genus-level diversity with dominant strains in the enrichments belonging to 12 different genera (Figure 3A and Supplementary Table 1). Most were classified as *Pseudomonas*, *Aeromonas*, *Polaromonas*, *Brevundimonas*, and *Acidovorax*, all of which feature species that have been detected in high-arsenic and/or low-temperature environments (Pepi et al., 2007; Paul et al., 2015; Singh et al., 2016; Ciok et al., 2018; Jiang et al., 2019). Eight fast-growing arsenic-tolerant microorganisms were isolated from the enrichments (Supplementary Table 4 and Supplementary Figure 1). The isolated strains were identified as *Pseudomonas* sp. (seven isolates) and *Rhodococcus* sp. (one isolate). Arsenic-tolerant microorganisms can promote plant growth and uptake of arsenic in arsenic-contaminated environments and are thus important in phytoremediation approaches (Mesa et al., 2017; Upadhyay et al., 2018). Some of the isolated strains from the present study might thus be used as inoculants to improve phytoremediation of arsenic-contaminated systems.

Dominant strains in arsenite-oxidizing enrichments were classified into eight different genera, the most abundant being *Pseudomonas*, *Polaromonas*, and *Ancylobacter* (Figure 3B and Supplementary Table 2). Arsenite oxidation has been reported for members of all three genera, and some *Polaromonas* are known to oxidize arsenite at low temperatures (Campos et al., 2010; Osborne et al., 2010, 2013; Andreoni et al., 2012). The five arsenite-oxidizing isolates were identified as belonging to the bacterial genera *Microbacterium* sp. (two isolates) and *Rhodococcus* sp. (two isolates) as well as to the fungal genus *Cadophora* sp. (one isolate) with sequence identities >99% to reference sequences (Supplementary Table 4 and Supplementary Figure 2). While *Microbacterium* spp. are known as arsenic-tolerant bacteria (see, e.g., Kaushik et al., 2012; Cordi et al., 2015; Dunivin et al., 2018), autotrophic arsenite oxidation has been shown only for one *Microbacterium maritypicum* strain (Li et al., 2016). Some strains of *Rhodococcus* are known to oxidize arsenite, and the genes encoding for arsenite oxidase have been detected in their genome (Heinrich-Salmeron et al., 2011; Kumari et al., 2019). No arsenite oxidation has been described for the fungal genus *Cadophora*. While fungal arsenite oxidation has been shown in a couple of strains (Ramos-Garza et al., 2016), research has largely been limited to bacterial arsenite oxidation, and further research is needed to gain better insight into fungal arsenite oxidation (Shi et al., 2020). Thus, the *Cadophora* sp. isolate obtained in the present study can be used in future studies on fungal arsenite oxidation.

Arsenate-respiring enrichments were likewise classified into eight genera, the majority being classified as *Rhodoferax* (>50% of all isolates) (Figure 3C). *Rhodoferax* strains possess *ars* genes involved in arsenate detoxification, tolerate arsenic in high concentrations, and have been detected in high-arsenic environments (Jackson et al., 2005; Giloteaux et al., 2013). However, *Rhodoferax* has not been reported to date as a dissimilatory arsenate reducer, even though strains of *Rhodoferax* are known for dissimilatory reduction of electron acceptors such as nitrate, manganese, or iron (Finneran et al., 2003). It is thus feasible that some *Rhodoferax* strains, including the strains enriched in the present study, might be capable of arsenate respiration. The remaining enriched arsenate-respiring microorganisms were identified as *Pseudomonas*, *Brevundimonas*, *Polaromonas*, or *Rahnella* (Figure 3C). Some species of *Pseudomonas*, *Brevundimonas*, and *Rahnella* have been shown to respire arsenate and to possess the *arrA* gene encoding for dissimilatory arsenate reductase (Youssef et al., 2008; Drewniak et al., 2014; Paul et al., 2015). Three fast-growing arsenate-respiring enrichments were isolated and identified as strains of *Pseudomonas* sp. with sequence identities of >99% to reference sequences (Supplementary Table 4 and Supplementary Figure 3). While it was desired to obtain *Rhodoferax* in pure culture for further physiological tests, the slow growth of the strains prevented complete isolation within the timeframe of the present study.

### Psychrotolerant Arsenic-Metabolizing Peat Microorganisms Are Well Adapted to *in situ* Temperatures

**FIGURE 4:**
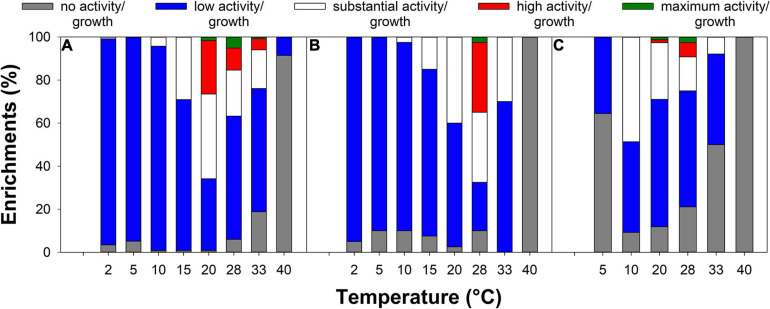
Effect of temperature on growth **(A)** or activity **(B,C)** of arsenic-tolerant **(A)**, arsenite-oxidizing **(B)**, and arsenate-respiring **(C)** enrichments. All observed growth and turnover rates were divided by the highest observed growth/turnover rate observed for the respective isolation approach and scored as follows: no observed activity/growth (0–2% of maximum), low activity/growth (2–24% of maximum), substantial activity/growth (25–49% of maximum), high activity/growth (50–74% of maximum), and maximum activity/growth (75–100% of maximum).

The highest arsenite oxidation activity was observed at 20–28°C for most arsenite-oxidizing enrichments (Figure 4B and Supplementary Table 2), while the highest arsenate reduction activity of many arsenate-respiring enrichments was observed at 10 or 20°C (Figure 4C and Supplementary Table 3). Most arsenic-tolerant enrichments showed the highest growth at 25–33°C (Figure 4A and Supplementary Table 1). The enrichments showed growth and activity in a range from 2 to 40°C (Figure 4 and Supplementary Tables 1–3). While only a few arsenic-tolerant enrichments showed growth at 40°C and none of the arsenite-oxidizing/arsenate-respiring enrichments showed activity at that temperature, 27 to 96% of the enrichments showed growth/activity at the lowest tested temperature (2 or 5°C; Figure 4). This indicates that most enrichments contained psychrotrophs/psychrotolerant microorganisms that show growth and activity at low temperatures, rather than true psychrophiles for which optimum growth is observed at temperatures ≤15°C (Russell et al., 1990; De Maayer et al., 2014). Microorganisms isolated from (seasonally) cold habitats are often psychrotrophs or even mesophiles (Master and Mohn, 1998). While psychrotolerants can be active at low temperatures, their metabolic rates are usually rather low and some might even enter a viable but non-culturable state during periods of cold temperature to be reactivated when temperatures are more favorable (De Maayer et al., 2014). The ability to withstand low temperatures is important for survival in (seasonally) cold habitats such as the Northern treatment peatlands from which the isolates originated, as winter peat temperatures can be close to or even below 0°C. However, it has to be noted that the isolation conditions with incubations at 10°C were likely not cold enough to select for true psychrophiles.

### Physiological Response of Arsenic-Metabolizing Microorganisms From Peat to Different Arsenic Species

**FIGURE 5:**
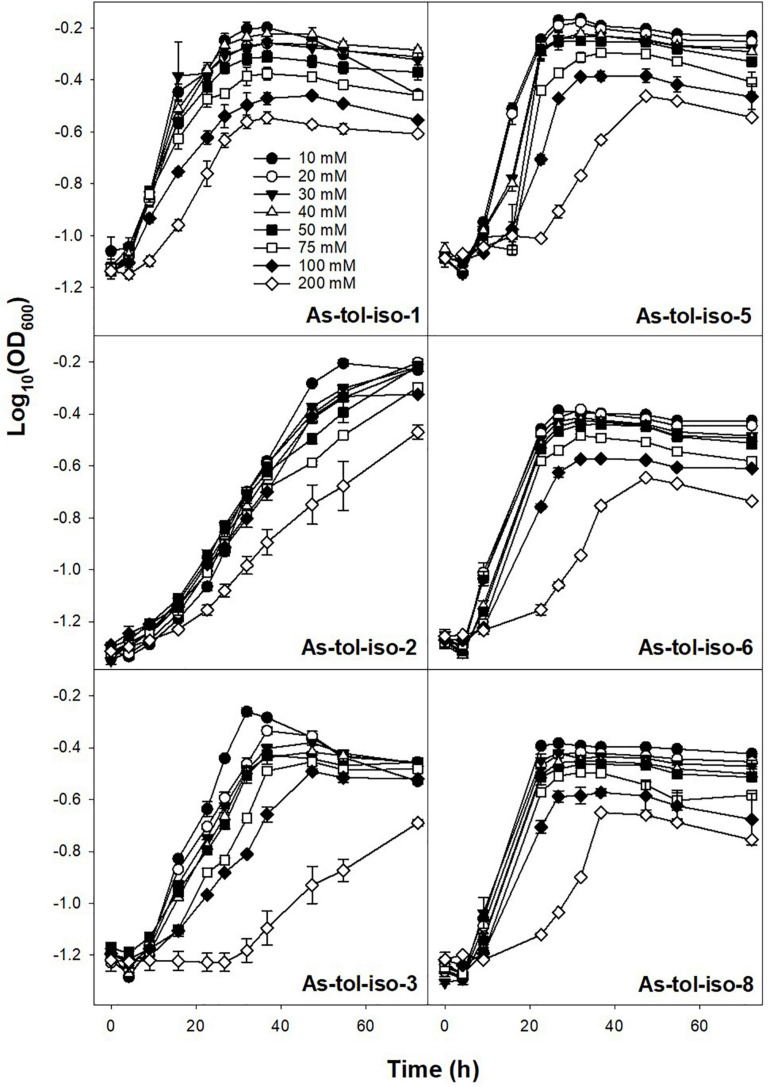
Effect of arsenate concentration on the growth of isolated arsenic-tolerant peat microorganisms. Strains were incubated in medium for arsenic-tolerant microorganisms supplemented with 10–200 mM arsenate and 10 mM arsenite at 20° in the dark. Mean values and standard errors of three replicates are shown.

**FIGURE 6:**
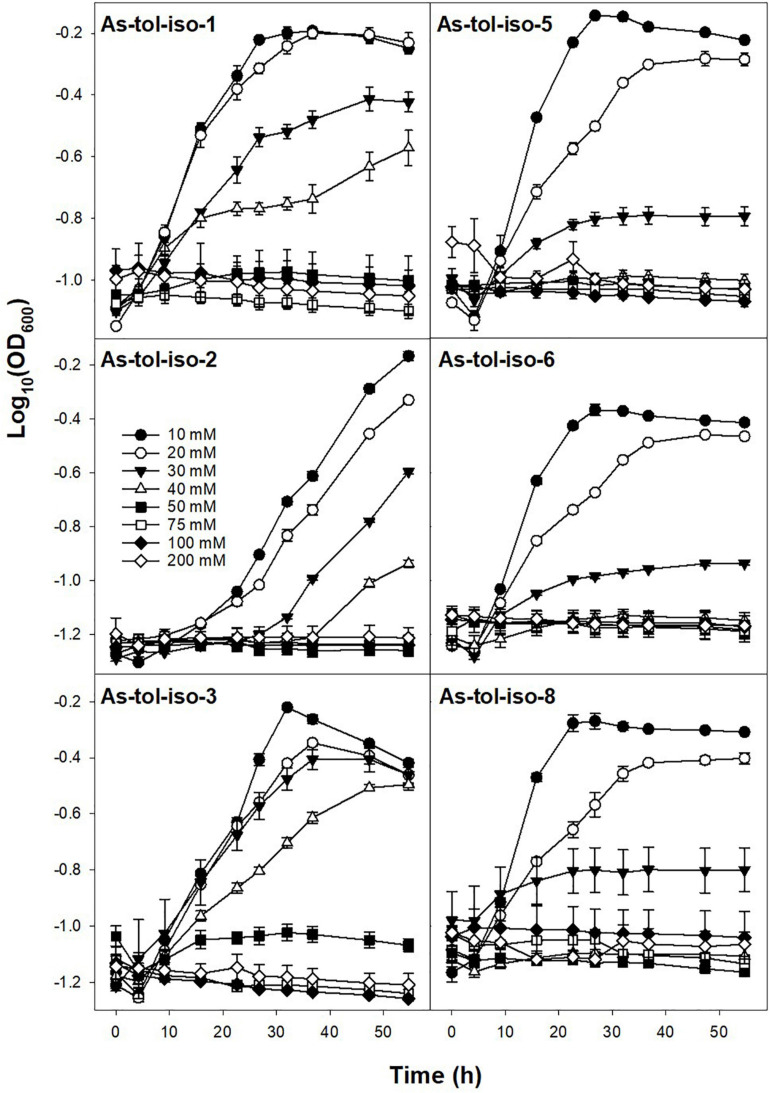
Effect of arsenite concentration on the growth of isolated arsenic-tolerant peat microorganisms. Strains were incubated in medium for arsenic-tolerant microorganisms supplemented with 10–200 mM arsenite and 10 mM arsenate at 20° in the dark. Mean values and standard errors of three replicates are shown.

Tolerance to arsenate or arsenite in concentrations ranging from 10 to 200 mM was tested for six out of eight arsenic-tolerant strains. All tested strains showed high tolerance to arsenate (Figure 5). While most strains showed lower growth rates and lower maximum OD at arsenate concentrations of 100 and 200 mM, growth was not completely inhibited by 200 mM arsenate. Tolerance to arsenite was much lower for all strains (Figure 6). For all but two strains, growth rates were slower at arsenite concentrations of 20 mM or higher, and complete inhibition of growth was observed at 40–50 mM for most strains. Only one strain showed marginal growth at arsenite concentrations of 50 mM. Arsenate tolerance has frequently been found to be higher than arsenite tolerance (e.g., Pepi et al., 2007; Dey et al., 2016; Dunivin et al., 2018). The reported minimum inhibitory concentrations of arsenic-resistant microorganisms are in the range of 60 to >300 mM for arsenate and in the range of <10–60 mM arsenite (e.g., Pepi et al., 2007; Kaushik et al., 2012; Dey et al., 2016; Dunivin et al., 2018), indicating that the strains isolated in the present study have a comparably high arsenic toleration and might thus be well suited for biotechnological applications in high-arsenic environments.

**FIGURE 7:**
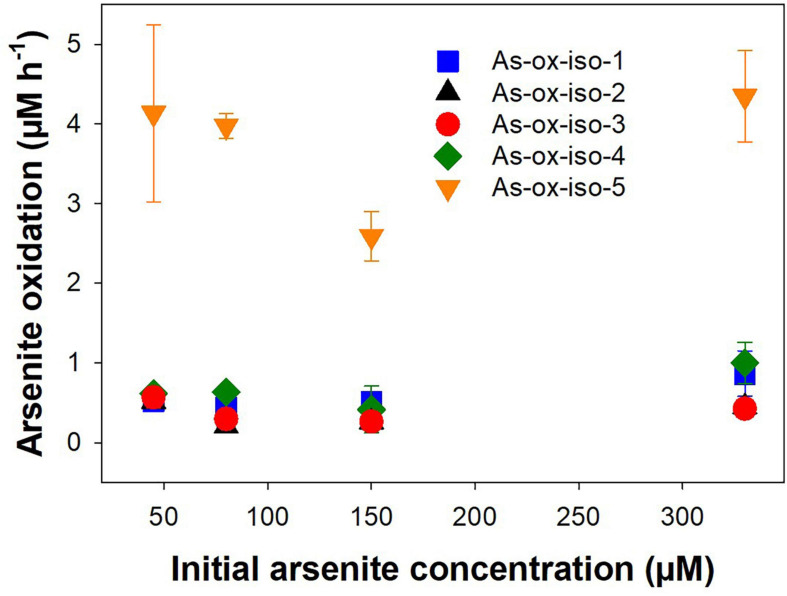
Effect of initial arsenite concentration on arsenite oxidation rates in arsenite-oxidizing microorganisms isolated from peat soil. Strains were incubated in arsenite oxidizer medium supplemented with 45–330 mM arsenite at 20° in the dark. Rates were normalized against the OD_660 *nm*_ of the first sampling timepoint. Mean values and standard errors of three replicates are shown.

Five autotrophic arsenite-oxidizing strains were tested for their arsenite oxidation potential at different initial arsenite concentrations ranging from 45 to 330 μM. The initial arsenite oxidation rates were linear and concomitant with an increase in OD (Supplementary Figure 4). Similar arsenite oxidation rates were observed for all tested initial arsenite concentrations (Figure 7), indicating that arsenite oxidation was not concentration-dependent in the tested range. All bacterial strains showed similar arsenite oxidation rates (on average 500 nM h^–1^), while the arsenite oxidation rates of the fungal isolate were higher (on average 4 μM h^–1^; Figure 7). In the treatment peatland, aerobic arsenite oxidation is likely the less important process since incoming arsenic is mainly in the form of arsenate, even though there is a rather high arsenite oxidation potential in peat soil (Kujala et al., 2020).

**FIGURE 8:**
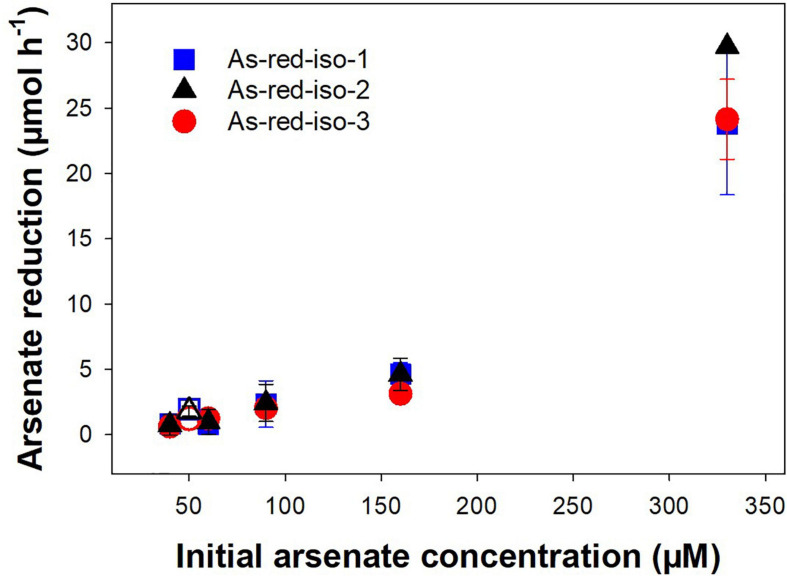
Effect of initial arsenate concentration on arsenate reduction rates in arsenate-respiring microorganisms isolated from peat soil. Strains were incubated in medium for arsenate-respiring microorganisms supplemented with 45 μM to 330 mM arsenate or artificial mine water supplemented with 50 μM arsenate at 20° in the dark. Rates were normalized against the OD_660 *nm*_ of the first sampling timepoint. Mean values and standard errors of three replicates are shown. Closed symbols = incubation in medium for arsenate-respiring microorganisms, open symbols = incubation in artificial mine water.

The three isolated *Pseudomonas* strains were tested for their arsenate reduction potential at different arsenate concentrations ranging from 45 to 330 μM. All three strains reduced arsenate, and arsenate reduction was concomitant with an increase in OD (Supplementary Figure 5). Observed arsenate reduction rates increased with increasing initial arsenate concentration and were similar for all three *Pseudomonas* strains (Figure 8). In the tested concentration range, the increase in arsenate reduction rate was nearly linear, and saturation of the process had likely not been achieved at the highest concentration. Arsenate reduction is an important process in the treatment peatland, converting the incoming arsenate to arsenite and thus facilitating its removal by binding to peat organic matter (Langner et al., 2012; Kujala et al., 2020). However, mine process water contains many other contaminants such as nitrogen compounds or sulfate in addition to arsenic, and arsenate reduction in the presence of these contaminants is thus crucial for arsenic removal. Strains incubated with artificial mine water containing 50 μM arsenate as well as sulfate, nitrate, and ammonium showed similar arsenate reduction capacities as strains incubated in arsenate respirer medium (Figure 8 and Supplementary Figure 5), indicating that indeed the presence of alternative electron acceptors such as nitrate and sulfate did not significantly reduce arsenate reduction rates.

### Low Abundance but High Functional Importance: Comparison of Culture-Dependent and Culture-Independent Diversity of Arsenic-Metabolizing Microorganisms in Peat

Enzymes involved in the transformation of arsenic are important for its turnover in the environment, and genes encoding these enzymes can thus be used as functional gene markers to detect potential arsenic-tolerant, arsenite-oxidizing, and arsenate-respiring microorganisms. Arsenic-tolerant microorganisms reduce arsenate to arsenite *via* the detoxifying arsenate reductase ArsC, while arsenate-respiring microorganisms reduce arsenate *via* the dissimilatory arsenate reductase ArrAB (Oremland and Stolz, 2003). The genes *arsC* and *arrA* are frequently used as functional gene markers for detoxifying and dissimilatory arsenate reduction, respectively (e.g., Malasarn et al., 2004; Escudero et al., 2013; Kujala et al., 2020). On the other hand, the arsenite oxidase AioAB catalyzes microbial arsenite oxidation (Oremland and Stolz, 2003) and the gene encoding its large subunit, *aioA*, has been used as a functional marker for arsenite oxidation (e.g., Inskeep et al., 2007; Escudero et al., 2013; Kujala et al., 2020).

**FIGURE 9:**
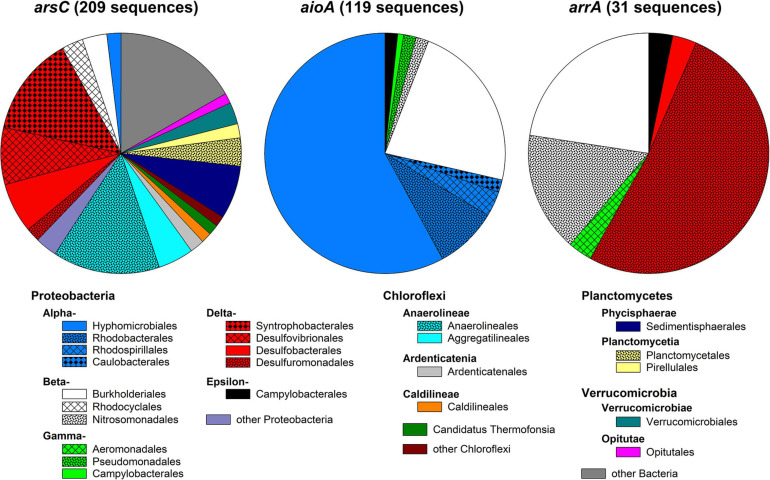
Order-level diversity of functional genes involved in arsenic metabolism in peat. Metagenomic reads were searched against custom databases for *arsC* (encoding the detoxifying arsenate reductase), *aioA* (encoding the arsenite oxidase), and *arrA* (encoding the dissimilatory arsenate reductase). Only matches with lengths ≥100 bp were used.

BLAST+ search of the metagenomic reads against custom *arsC*, *aioA*, and *arrA* databases detected 209, 119, and 31 matches with sequence lengths of 100 bp or longer. Detected *arsC* were often only distantly related to *arsC* of pure cultures (on average 76% sequence identity; Supplementary Table 5), while detected *aioA* and *arrA* were more closely related to those of pure cultures (on average 95 and 90% sequence identity, respectively; Supplementary Tables 6, 7). Detected *arsC* were affiliated to 75 bacterial and archaeal genera (Supplementary Table 5) belonging to the Deltaproteobacterial orders Syntrophobacterales, Desulfovibrionales, and Desulfobacterales; the orders Anaerolineales and Aggregatilneales of the Chloroflexi; and other Proteobacteria, Planctomycetes, and Verrucomicrobia (Figure 9). The most abundant genera were *Syntrophus* (9.6% of detected *arsC*), *Anaerolinea* (7.2% of detected *arsC*), and *Sedimentisphaera* (5.7% of detected *arsC*). Detected *aioA* were affiliated to 58 bacterial genera which mainly belonged to the Alphaproteobacterial orders Hyphomicrobiales and Rhodobacterales as well as the Betaproteobacterial order Burkholderiales (Figure 9). The most abundant genera were *Mesorhizobium* (11% of detected *aioA*), *Acidovorax* (4.2% of detected *aioA*), *Ciceribacter* (4.2% of detected *aioA*), and *Rhodoferax* (3.4% of detected *aioA*; Supplementary Table 6). *arrA* detected in the metagenomic reads were affiliated to six bacterial genera mainly belonging to the Betaproteobacteria (orders Burkholderiales and Nitrosomonadales) and Deltaproteobacteria (orders Desulfuromonadales and Desulfobacterales; Figure 9). Most *arrA* were affiliated to *Geobacter* (52%), *Rubrivivax* (23%), and *Sulfuritalea* (16%; Supplementary Table 7). This indicates a pronounced genetic potential for arsenic turnover in the peatland, and arsenic turnover has indeed been demonstrated in incubations with peatland soil previously (Kujala et al., 2020). Amplicon-based studies with peat from the same site likewise detected the presence of *Syntrophus*- and *Sedimentisphaera*-affiliated *arsC* as well as *Geobacter*- and *Sulfuritalea*-affiliated *arrA* (Kujala et al., 2020). Apart from *Rhodoferax*, most of the abundant genera indicated in the functional gene analysis were not detected in the culture-dependent approach, indicating that the cultivation detected different arsenic-metabolizing microorganisms than the metagenome analysis. This could be due on the one hand to selective conditions in the isolation approaches, on the other hand to bias in the databases used to search for arsenic-metabolism genes in the metagenome. Moreover, it should be kept in mind that the identity of the detected genes with genes from pure cultures was often low (especially for *arsC*); hence, the functional gene markers cannot be used for exact taxonomic identification.

Even though they were not among the abundant genera, several of the isolated and enriched genera were nonetheless detected based on the taxonomic annotation of the metagenome. Of the isolated genera, *Pseudomonas* had a relative abundance of 0.8% in the metagenomic reads, while *Rhodococcus* had a relative abundance of 0.3% based on RefSeq annotation. *Microbacterium* was not detected in RefSeq annotated reads but based on the Silva SSU annotation of the rRNA genes (0.05% of all 16S rRNA reads from the shotgun metagenome). Matches for all isolates and most of the dominant strains in the enrichments were detected in the metagenomic reads using BLAST+ (Supplementary Table 4). While those reads were short and did not cover the whole 16S rRNA gene, the presence of the isolated strains in the peat metagenome is indicated in low abundance. Microorganisms with low abundance in an environment (or the “rare biosphere”) often have high functional potential and phylogenetic diversity (Lynch and Neufeld, 2015) and may moreover play an important role in ecosystem functioning (Jousset et al., 2017). Thus, even though the enriched and isolated genera may not be the most abundant ones *in situ*, they might still contribute to arsenic cycling in treatment peatlands. Moreover, their arsenic-metabolism genes might not be well represented in currently available databases, thus emphasizing the need for further studies on the isolates in respect to their arsenic metabolism. The exploitation of rare biosphere microorganisms for biotechnological applications such as bioremediation has been suggested (Pascoal et al., 2020); thus, enrichments and isolates from the present study should be further tested for their bioremediation potential.

### Evaluation of the Dilution-to-Extinction Approach for Isolation of Arsenic-Metabolizing Microorganisms From Peat

The dilution-to-extinction approach used in this study revealed a rather limited diversity of arsenic-tolerant, arsenite-oxidizing, and arsenate-respiring microorganisms with Shannon diversities of 1.7, 1.6, and 0.8, respectively (Table 2). Especially for the arsenate-respiring microorganisms, the dominant strains in most enrichments were rather closely related to each other and were classified into a single genus, *Rhodoferax*, which was also reflected by a rather low evenness of 0.2 (Supplementary Table 2). This indicates a strong predominance of *Rhodoferax*-related culturable arsenate-respiring microorganisms in the peat. Even though the dilution-to-extinction approach does not enrich for less abundant but faster-growing microorganisms, the obtained diversity may nonetheless be limited due to the occurrence of some dominant taxa in uneven microbial communities. The dominant taxa detected in the cultivation approach differed strongly from those detected in earlier studies from the same peatland which targeted general and arsenic-metabolizing microbial communities using amplicon sequencing of 16S rRNA genes and functional genes involved in arsenic turnover, respectively (Kujala et al., 2018, 2020), as well as from the dominant taxa in the metagenome. Many studies that compared culture-dependent and culture-independent approaches find that different fractions of the microbial community are detected with either approach (e.g., Vaz-Moreira et al., 2011; Shade et al., 2012; Stefani et al., 2015), but results from culture-dependent and -independent approaches are nonetheless equally valuable.

*Geobacter*-related isolates were not obtained, even though this genus was rather abundant based on the metagenomic reads and could have been expected to grow in the approach targeting arsenic-tolerating or arsenate-respiring microorganisms. One reason for this is likely the selective nature of the isolation approaches (specific growth medium, growth temperature, etc.), and modifications to the isolation setup might thus increase the diversity of the obtained strains. It is well known that the majority of environmental microorganisms cannot be easily cultivated under laboratory conditions, and more sophisticated approaches might be needed for their cultivation (see, e.g., Stewart, 2012). Information about metabolic potential and environmental requirements of uncultured microorganisms gained from metagenomic analyses can be used to optimize future cultivation efforts (Lagier et al., 2016; Gutleben et al., 2018).

## Conclusion and Future Steps

More than 200 arsenic-metabolizing enrichments belonging to 20 genera and 16 pure cultures were obtained in the present study. While many of these genera have been previously reported to be arsenic-tolerant, arsenite-oxidizing, or arsenate-respiring, some were classified as genera that are not yet known to have these metabolic capabilities. The further study of the enriched strains, their metabolic pathways, and the genes involved in arsenic turnover will thus be of great interest. After additional steps have been taken to ensure the pure culture status of the remaining enrichments, the most interesting strains (e.g., *Rhodoferax*-related arsenate respirers) will be characterized in more detail and will (in part) serve as model organisms for further studies of arsenic-metabolizing microbial activities in peatlands. Characterization of the strains should include (i) further physiological experiments, (ii) genome sequencing and annotation for the isolates of interest, leading to identification of genes and pathways potentially involved in arsenic turnover, (iii) expression studies under a variety of environmental conditions to elucidate which of the identified genes and pathways are turned on for arsenic metabolism and which parameters stimulate or repress their expression, and (iv) further efforts to elucidate the potential of the organisms for arsenic bioremediation in larger scale water treatment solutions.

## Data Availability Statement

The original contributions presented in the study are publicly available. 16S rRNA gene and ITS sequence data can be found in NCBI under accession numbers MW496900–MW497062 (enrichments), MW800181–MW800195 (bacterial isolates), and MW800602 (fungal isolate). The metagenome and metatranscriptome sequence data are available at MGRast under project numbers mgm4838788.3, mgm4928889.3, and mgm4928890.3.

## Author Contributions

Both authors contributed to the experimental design, laboratory experiments, and data collection as well as data analysis, and revised the manuscript. KK wrote the main manuscript text.

## Conflict of Interest

The authors declare that the research was conducted in the absence of any commercial or financial relationships that could be construed as a potential conflict of interest.

## Footnotes

1 www.arb-silva.de
