# Supplementary material for: Assessing the Diversity and Metabolic Potential of Psychrotolerant Arsenic-Metabolizing Microorganisms From a Subarctic Peatland Used for Treatment of Mining-Affected Waters by Culture-Dependent and -Independent Techniques
Source: Front Microbiol. 2021 Jul 6;12:648412. doi: 10.3389/fmicb.2021.648412 (PMC8290898; doi:10.3389/fmicb.2021.648412)
Supplement: Supplementary file 1 [file Data_Sheet_1.pdf]

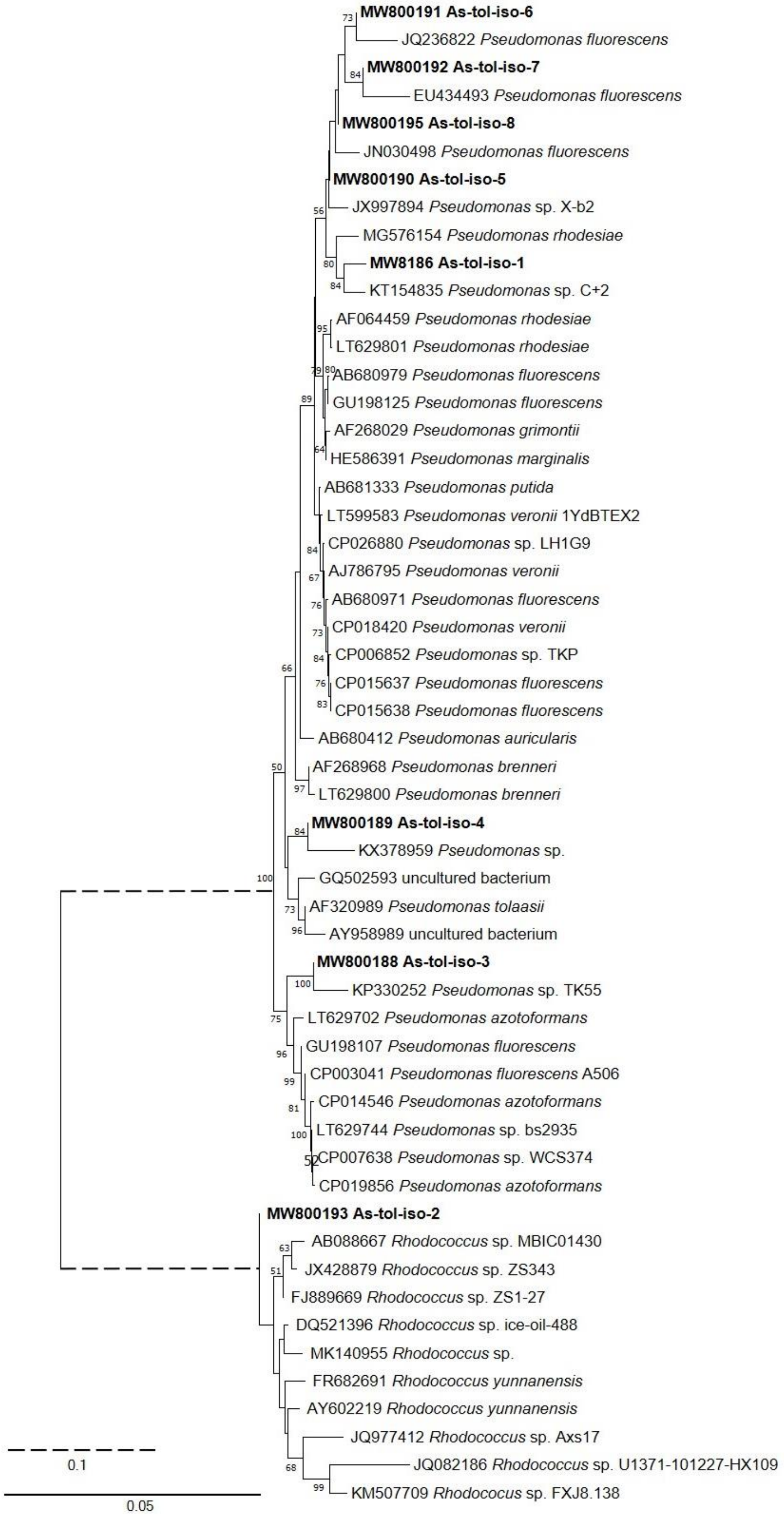

Figure S1: Phylogenetic tree of 16S rRNA gene sequences obtained from arsenic-tolerant strains. Reference sequences from cultured species and uncharacterized microorganisms were obtained from public databases. Neighbor Joining trees were constructed in MEGA 7. Bootstrap values (1000 replications) are indicated next to the branches, bootstrap values < 50% have been omitted.

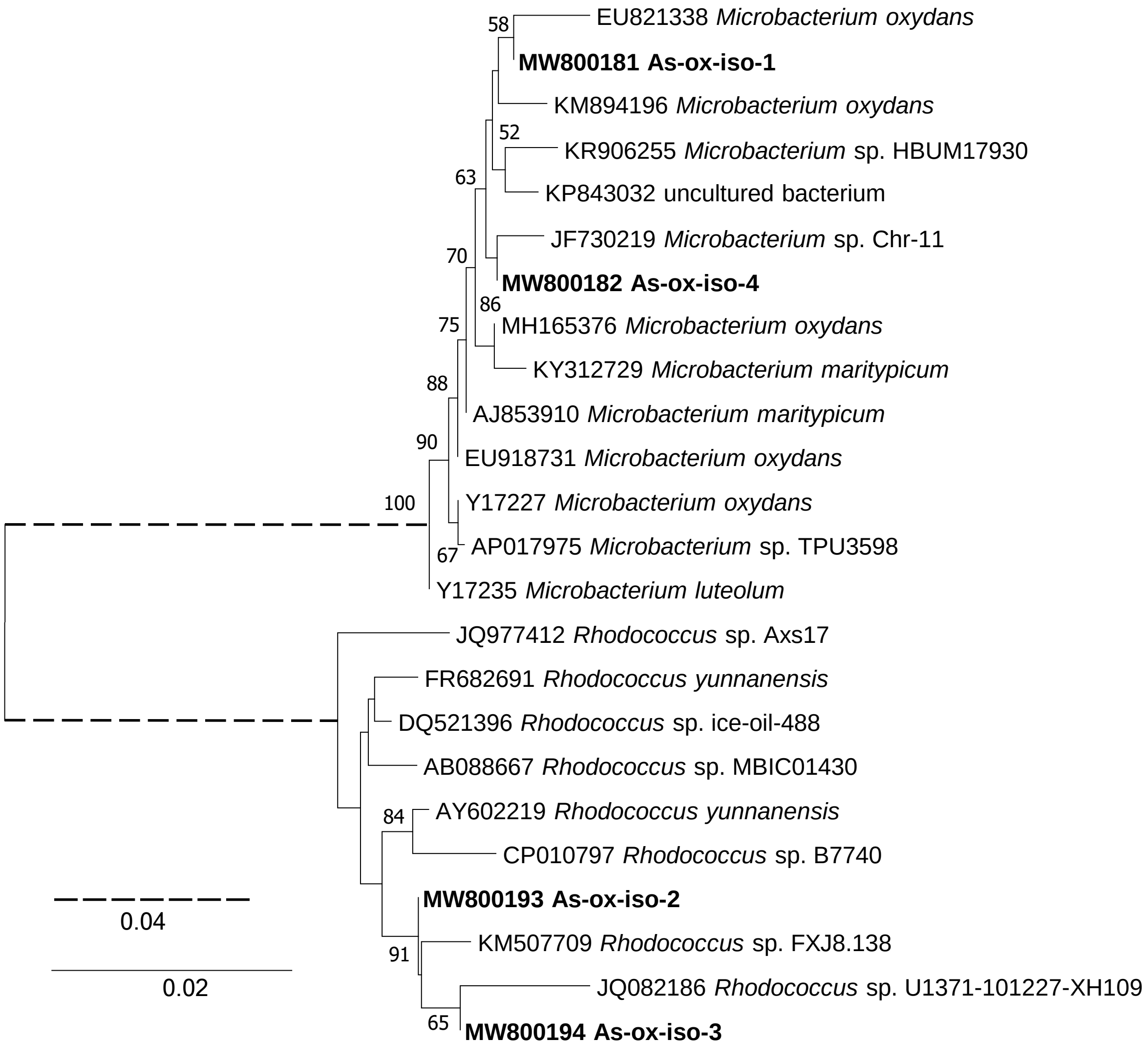

Figure S2: Phylogenetic tree of 16S rRNA gene sequences obtained from bacterial arsenite-oxidizing strains. Reference sequences from cultured species and uncultured microorganisms were obtained from public databases. Neighbor Joining trees were constructed in MEGA 7. Bootstrap values (1000 replications) are indicated next to the branches, bootstrap values < 50% have been omitted.

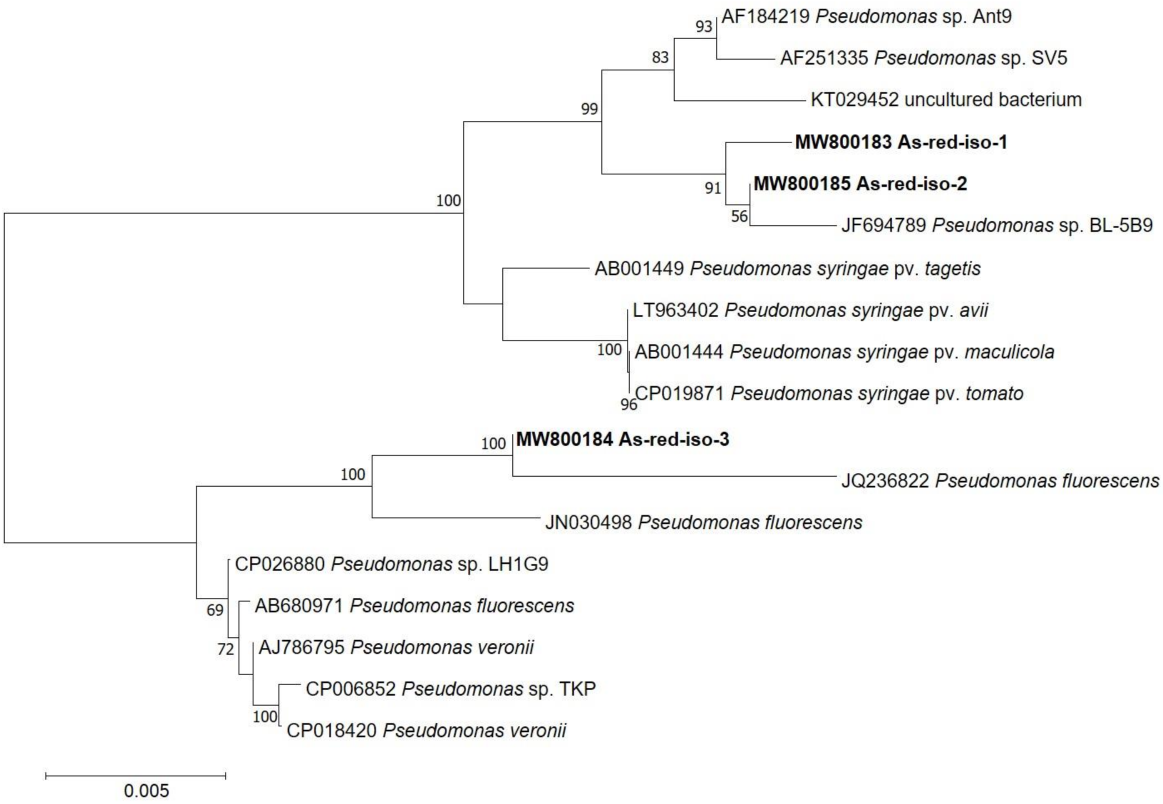

Figure S3: Phylogenetic tree of 16S rRNA gene sequences obtained from arsenate-respiring strains. Reference sequences from cultured species and uncharacterized microorganisms were obtained from public databases. Neighbor Joining trees were constructed in MEGA 7. Bootstrap values (1000 replications) are indicated next to the branches, bootstrap values < 50% have been omitted.

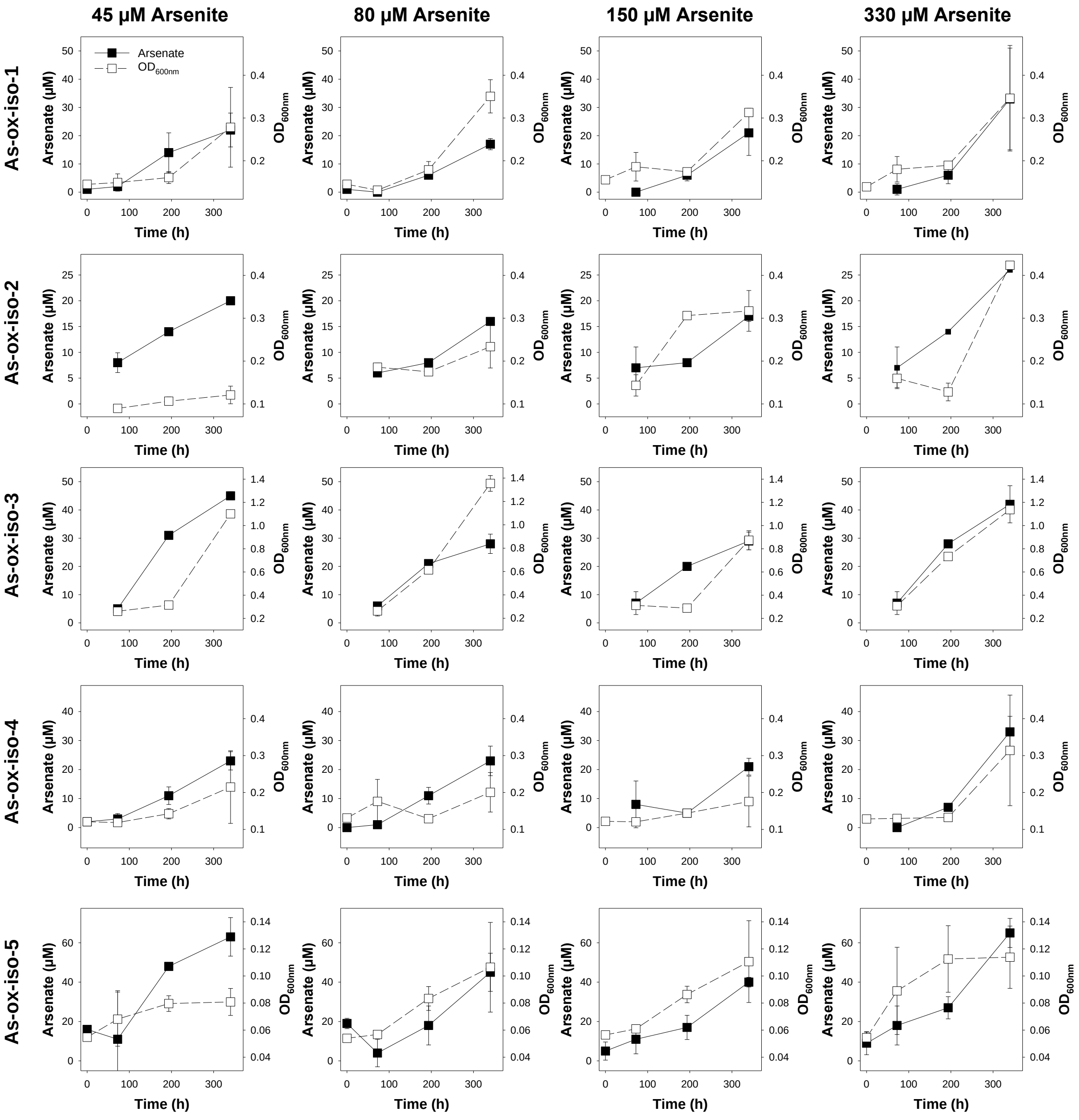

Figure S4: Arsenite oxidation and microbial growth in incubations supplemented with different initial arsenite concentrations. Mean values and standard errors of three replicates are shown.

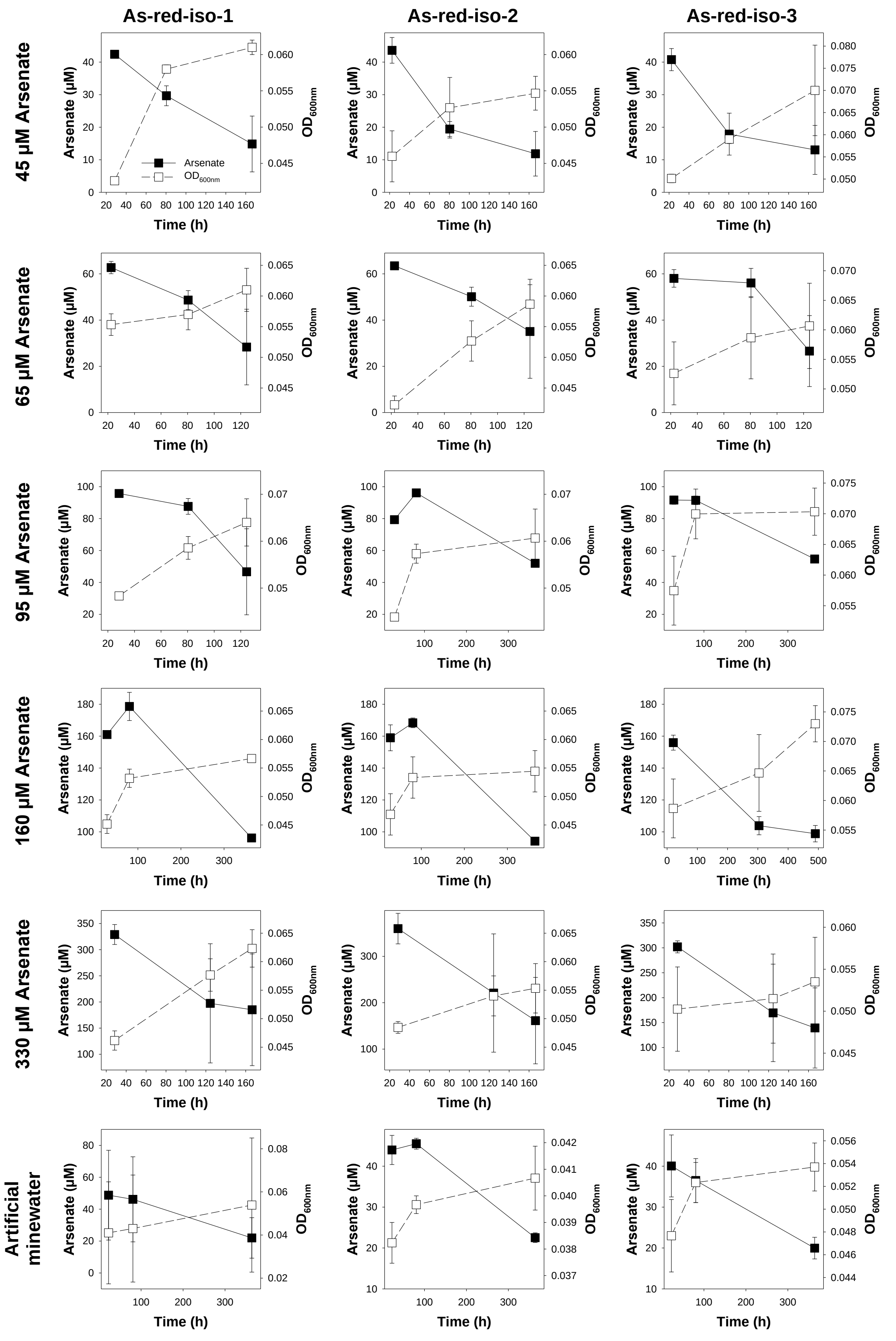

Figure S5: Arsenate reduction and microbial growth in incubations supplemented with different initial arsenate concentrations. Mean values and standard errors of three replicates are shown.

Table S1: Identification and characterization of arsenic-tolerant microorganisms from peat. Enrichments were classified based on the sequence of the dominant strain using the SILVA Alignment, Classification and Tree Service and most closely related 16S rRNA gene reference sequences were identified using BLAST. Enrichments which were further purified to obtain pure culture strains are marked in bold. Growth at different temperatures is given in categories relative to the highest observed growth rate: (-) no observed growth (0-2% of maximum), (+) low growth (2-24% of maximum), (++) substantial growth (25-49% of maximum), (+++) high growth (50-74% of maximum), and (++++) maximum growth (75-100% of maximum).

Enrichment name	Sequencing results: Silva classification					Sequencing results: Next related sequence			Potential pure culture [a]	Growth observed at							
	Phylum	Class	Order	Family	Genus	Accession no.	Name	% similarity		2 ºC	5 ºC	10 ºC	20 ºC	25 ºC	28 ºC	33 ºC	40 ºC
As-Tol1				sequencing not successful						+	+	+	+	+	+	-	-
**As-Tol2**	**Proteobacteria**	**Gammaproteobacteria**	**Xanthomonadales**	**Rhodanobacteraceae**	***Rhodanobacter***	**MUNR01000001**	***Rhodanobacter* sp. C01**	**88.3**	**-**	**+**	**+**	**+**	**+**	**++**	**+**	**+**	**-**
As-Tol3	Proteobacteria	Gammaproteobacteria	Pseudomonadales	Pseudomonadaceae	*Pseudomonas*	D86003	*Pseudomonas fluorescens*	98.9	+	+	+	+	+	+	++	-	-
As-Tol4	Proteobacteria	Gammaproteobacteria	Pseudomonadales	Pseudomonadaceae	*Pseudomonas*	EU434397	*Pseudomonas fluorescens*	98.7	+	+	+	+	++	+++	++++	++	-
As-Tol5	Proteobacteria	Gammaproteobacteria	Pseudomonadales	Pseudomonadaceae	*Pseudomonas*	EU434397	*Pseudomonas fluorescens*	98.7	+	+	+	+	+	+++	+++	++	-
As-Tol6	Proteobacteria	Gammaproteobacteria	Pseudomonadales	Pseudomonadaceae	*Pseudomonas*	CP010945	*Pseudomonas fluorescens* NCIMB 11764	99.3	+	+	+	+	+	++	+++	+	-
As-Tol7	Proteobacteria	Gammaproteobacteria	Pseudomonadales	Pseudomonadaceae	*Pseudomonas*	JF694789	*Pseudomonas* sp. BLH-5B9	99.2	-	+	+	+	++	++	++	+	-
As-Tol8	Proteobacteria	Gammaproteobacteria	Pseudomonadales	Pseudomonadaceae	*Pseudomonas*	CP010945	*Pseudomonas fluorescens* NCIMB 11764	98.3	+	+	+	+	+	+	+	-	-
As-Tol9	Proteobacteria	Alphaproteobacteria	Caulobacterales	Caulobacteraceae	*Brevundimonas*	JN009622	*Brevundimonas* sp. GC044	95.7	-	+	+	+	+	+	++	+	-
As-Tol10	Proteobacteria	Gammaproteobacteria	Enterobacterales	Aeromonadaceae	*Aeromonas*	JX872320	uncultured bacterium	98.8	+	+	+	+	++	+	+	-	-
As-Tol11				sequencing not successful						+	+	+	+	+	+	+	-
As-Tol12				sequencing not successful						+	+	+	+	+	+	+	-
As-Tol13	Proteobacteria	Gammaproteobacteria	Enterobacterales	Aeromonadaceae	*Aeromonas*	JX872320	uncultured bacterium	98.8	+	+	+	+	+	++	++	+	-
As-Tol14	Proteobacteria	Gammaproteobacteria	Enterobacterales	Aeromonadaceae	*Aeromonas*	JX872320	uncultured bacterium	98.8	+	+	+	+	+	+++	+++	+++	-
As-Tol15	Proteobacteria	Gammaproteobacteria	Pseudomonadales	Pseudomonadaceae	*Pseudomonas*	JF694789	*Pseudomonas* sp. BLH-5B9	98.3	+	+	+	+	++	+	+	+	-
As-Tol16	Proteobacteria	Gammaproteobacteria	Pseudomonadales	Pseudomonadaceae	*Pseudomonas*	D86003	*Pseudomonas fluorescens*	97.8	-	+	+	+	++	+++	++	++	-
As-Tol17	Proteobacteria	Gammaproteobacteria	Pseudomonadales	Pseudomonadaceae	*Pseudomonas*	JX997894	*Pseudomonas* sp. X-b2	97.1	+	+	+	+	++	+++	++++	+	-
As-Tol18				sequencing not successful						+	+	+	+	++	++	+	-
As-Tol19	Proteobacteria	Gammaproteobacteria	Enterobacterales	Aeromonadaceae	*Aeromonas*	JX872320	uncultured bacterium	99.3	-	+	+	+	++	+++	+++	++	-
As-Tol20	Proteobacteria	Gammaproteobacteria	Enterobacterales	Aeromonadaceae	*Aeromonas*	JX872320	uncultured bacterium	99.3	-	+	+	+	+	+	+	+	-
As-Tol21	Proteobacteria	Alphaproteobacteria	Caulobacterales	Caulobacteraceae	*Brevundimonas*	JN009622	*Brevundimonas* sp. GC044	95.7	+	+	+	+	+	+	+	+	-
As-Tol22	Proteobacteria	Gammaproteobacteria	Enterobacterales	Aeromonadaceae	*Aeromonas*	JX872320	uncultured bacterium	99.3	-	+	+	+	++	+++	++++	+++	-
As-Tol23	Proteobacteria	Gammaproteobacteria	Enterobacterales	Aeromonadaceae	*Aeromonas*	JX872320	uncultured bacterium	98.8	+	+	+	+	++	+++	+++	++++	-
As-Tol24	Proteobacteria	Gammaproteobacteria	Burkholderiales	Comamonadaceae	*Polaromonas*	KJ615244	uncultured bacterium	94.1	+	+	+	+	+	++	+++	+	-
As-Tol25	Proteobacteria	Gammaproteobacteria	Pseudomonadales	Pseudomonadaceae	*Pseudomonas*	FJ984605	*Pseudomonas* sp. gvg9	96.6	-	+	+	+	+	+	+	+	-
As-Tol26				sequencing not successful						+	+	+	+	++	+	+	-
As-Tol27	Proteobacteria	Gammaproteobacteria	Pseudomonadales	Pseudomonadaceae	*Pseudomonas*	JF901709	*Pseudomonas* sp. A-BT-68	98.5	-	+	+	+	+	++	+	+	-
As-Tol28	Proteobacteria	Gammaproteobacteria	Enterobacterales	Aeromonadaceae	*Aeromonas*	JX872320	uncultured bacterium	98.8	-	+	+	+	++	+++	++	+++	-
As-Tol29	Proteobacteria	Gammaproteobacteria	Pseudomonadales	Pseudomonadaceae	*Pseudomonas*	KJ534472	*Pseudomonas rhodesiae*	97.9	+	+	+	+	++	+++	++	++	-
As-Tol30				sequencing not successful						+	+	++	++	+++	++++	++	-
As-Tol31	Proteobacteria	Gammaproteobacteria	Enterobacterales	Aeromonadaceae	*Aeromonas*	FR853560	uncultured bacterium	92.3	-	+	+	++	++	+	-	+	-
As-Tol32				sequencing not successful						+	+	++	++	+++	++++	+++	-
As-Tol33	Proteobacteria	Gammaproteobacteria	Enterobacterales	Aeromonadaceae	*Aeromonas*	CP017143	*Aeromonas salmonicida* subsp. *masoucida*	95.8	-	+	+	+	++	+++	++	+++	-
As-Tol34	Proteobacteria	Gammaproteobacteria	Pseudomonadales	Pseudomonadaceae	*Pseudomonas*	CP010945	*Pseudomonas fluorescens* NCIMB 11764	99.3	-	+	+	+	++	++	++	+	-
As-Tol35	Proteobacteria	Gammaproteobacteria	Burkholderiales	Comamonadaceae	*Polaromonas*	KJ615244	uncultured bacterium	94.1	+	+	+	+	+	+	+	+	-
As-Tol36	Proteobacteria	Gammaproteobacteria	Enterobacterales	Aeromonadaceae	*Aeromonas*	JX872320	uncultured bacterium	98.8	+	+	+	+	+	+	++	+	-
As-Tol37	Proteobacteria	Gammaproteobacteria	Enterobacterales	Aeromonadaceae	*Aeromonas*	JX872320	uncultured bacterium	98.8	+	+	+	+	+	++	+	+++	-
As-Tol38	Proteobacteria	Gammaproteobacteria	Pseudomonadales	Pseudomonadaceae	*Pseudomonas*	JQ995149	*Pseudomonas* sp. SJTE-2	98.0	+	+	+	+	+	++	+	+	-
As-Tol39	Proteobacteria	Gammaproteobacteria	Pseudomonadales	Pseudomonadaceae	*Pseudomonas*	JX997894	*Pseudomonas* sp. X-b2	97.1	+	+	+	+	+	+++	+	+	-
As-Tol40	Proteobacteria	Gammaproteobacteria	Pseudomonadales	Pseudomonadaceae	*Pseudomonas*	MH197384	*Pseudomonas gessardii*	97.1	+	+	+	+	++	+++	++++	++	-
**As-Tol41**	**Proteobacteria**	**Gammaproteobacteria**	**Enterobacterales**	**Aeromonadaceae**	***Aeromonas***	**KX344025**	***Aeromonas popoffii***	**78.8**	**-**	**+**	**+**	**+**	**++**	**+++**	**+++**	**+**	**-**
As-Tol42	Proteobacteria	Gammaproteobacteria	Pseudomonadales	Pseudomonadaceae	*Pseudomonas*	CP026880	*Pseudomonas* sp. LH1G9	98.8	+	+	+	+	++	+++	++	++	-
As-Tol43				sequencing not successful						+	+	+	+	+	+	+	-
As-Tol44	Proteobacteria	Gammaproteobacteria	Enterobacterales	Aeromonadaceae	*Aeromonas*	JX872320	uncultured bacterium	99.3	+	+	+	+	+	++	+	+	-
As-Tol45	Proteobacteria	Gammaproteobacteria	Burkholderiales	Comamonadaceae	*Polaromonas*	KJ615244	uncultured bacterium	98.2	-	+	+	+	+	++	+	++	-
As-Tol46	Proteobacteria	Alphaproteobacteria	Caulobacterales	Caulobacteraceae	*Brevundimonas*	JN030509	uncultured bacterium	92.5	-	+	+	+	+	+++	++	+	-
As-Tol47	Proteobacteria	Gammaproteobacteria	Burkholderiales	Comamonadaceae	*Acidovorax*	GU385755	uncultured bacterium	96.6	+	+	+	+	+	+	+	+	-
As-Tol48				sequencing not successful						+	+	+	+	+	++	+	-
As-Tol49	Proteobacteria	Gammaproteobacteria	Enterobacterales	Aeromonadaceae	*Aeromonas*	CP027000	*Aeromonas salmonicida* subsp. *salmonicida* 0	99.1	-	+	-	+	+	++	+	+	-
As-Tol50	Proteobacteria	Gammaproteobacteria	Pseudomonadales	Pseudomonadaceae	*Pseudomonas*	JF901709	*Pseudomonas* sp. A-BT-68	98.5	-	+	+	+	+	++	++	+	-
As-Tol51	Proteobacteria	Gammaproteobacteria	Pseudomonadales	Pseudomonadaceae	*Pseudomonas*	CP026880	*Pseudomonas* sp. LH1G9	99.0	+	+	+	+	++	+++	++	+	-
As-Tol52	Proteobacteria	Gammaproteobacteria	Xanthomonadales	Xanthomonadaceae	*Thermomonas*	FPLS01027968	metagenome	98.5	+	+	+	+	+	+	+	+	-
**As-Tol53**	**Proteobacteria**	**Gammaproteobacteria**	**Xanthomonadales**	**Xanthomonadaceae**	***Thermomonas***	**FPLS01027968**	**metagenome**	**98.5**	**-**	**+**	**+**	**+**	**++**	**++**	**+**	**+**	**-**
As-Tol54	Proteobacteria	Gammaproteobacteria	Pseudomonadales	Pseudomonadaceae	*Pseudomonas*	CP026880	*Pseudomonas* sp. LH1G9	98.8	+	+	+	+	++	+++	+++	++	-
As-Tol55	Proteobacteria	Gammaproteobacteria	Pseudomonadales	Pseudomonadaceae	*Pseudomonas*	CP026880	*Pseudomonas* sp. LH1G9	98.8	+	+	+	+	+	+++	+	++	+
As-Tol56	Proteobacteria	Alphaproteobacteria	Caulobacterales	Caulobacteraceae	*Brevundimonas*	JN030509	uncultured bacterium	88.0	-	+	+	+	+	+	+	++	-
As-Tol57	Proteobacteria	Gammaproteobacteria	Burkholderiales	Comamonadaceae	*Polaromonas*	KJ615244	uncultured bacterium	98.2	+	+	+	+	+	+	-	+	-
As-Tol58	Proteobacteria	Gammaproteobacteria	Burkholderiales	Comamonadaceae	*Acidovorax*	GU385755	uncultured bacterium	97.5	+	+	+	+	+	+	+	+	-
As-Tol59				sequencing not successful						+	+	+	+	++	+	+	-
As-Tol60	Proteobacteria	Alphaproteobacteria	Caulobacterales	Caulobacteraceae	*Asticcacaulis*	JX079513	uncultured *Asticcacaulis* sp.	97.2	-	+	+	+	+	+	++	+	-
**As-Tol61**	**Proteobacteria**	**Gammaproteobacteria**	**Pseudomonadales**	**Pseudomonadaceae**	***Pseudomonas***	**HF913576**	***Pseudomonas fluorescens***	**97.0**	**+**	**+**	**+**	**+**	**++**	**++**	**++**	**++**	**-**
As-Tol62	Proteobacteria	Gammaproteobacteria	Pseudomonadales	Pseudomonadaceae	*Pseudomonas*	CP026880	*Pseudomonas* sp. LH1G9	98.6	+	+	+	++	++	++++	+++	++	-
As-Tol63	Proteobacteria	Gammaproteobacteria	Pseudomonadales	Pseudomonadaceae	*Pseudomonas*	JX997894	*Pseudomonas* sp. X-b2	97.1	+	+	+	+	+	++	+	+	-
As-Tol64	Proteobacteria	Gammaproteobacteria	Pseudomonadales	Pseudomonadaceae	*Pseudomonas*	CP010945	*Pseudomonas fluorescens* NCIMB 11764	99.4	+	+	+	+	+	++	-	+	-
As-Tol65	Proteobacteria	Gammaproteobacteria	Pseudomonadales	Pseudomonadaceae	*Pseudomonas*	AMZG01000050	*Pseudomonas fluorescens* BS2	93.1	-	++	+	+	+	+++	++	+	-
As-Tol66	Proteobacteria	Gammaproteobacteria	Pseudomonadales	Pseudomonadaceae	*Pseudomonas*	D86003	*Pseudomonas fluorescens*	96.5	+	+	+	+	++	+++	+++	++	-
As-Tol67	Proteobacteria	Gammaproteobacteria	Pseudomonadales	Pseudomonadaceae	*Pseudomonas*	CP026880	*Pseudomonas* sp. LH1G9	98.8	+	+	+	+	++	+++	+++	++	-
As-Tol68	Proteobacteria	Alphaproteobacteria	Caulobacterales	Caulobacteraceae	*Brevundimonas*	JN009622	*Brevundimonas* sp. GC044	95.7	-	+	+	+	+	+	+	+	-
**As-Tol69**	**Actinobacteriota**	**Actinobacteria**	**Corynebacteriales**	**Nocardiaceae**	***Rhodococcus***	**JX105591**	**uncultured bacterium**	**99.5**	**-**	**+**	**+**	**+**	**+**	**+**	**+**	**+**	**-**
**As-Tol70**	**Actinobacteriota**	**Actinobacteria**	**Micrococcales**	**Microbacteriaceae**	***Cryobacterium***	**CP016282**	***Cryobacterium arcticum***	**98.1**	**-**	**+**	**+**	**+**	**+**	**+**	**+**	**+**	**-**
As-Tol71	Proteobacteria	Alphaproteobacteria	Caulobacterales	Caulobacteraceae	*Asticcacaulis*	JX079513	uncultured *Asticcacaulis* sp.	98.7	+	+	-	+	+	+	+	+	-
**As-Tol72**				**sequencing not successful**						**+**	**+**	**+**	**+**	**++**	**+**	**+**	**-**
As-Tol73	Proteobacteria	Gammaproteobacteria	Burkholderiales	Comamonadaceae	*Polaromonas*	KJ615244	uncultured bacterium	94.1	+	+	+	+	+	+	+	-	-
As-Tol74	Proteobacteria	Gammaproteobacteria	Pseudomonadales	Pseudomonadaceae	*Pseudomonas*	KP230457	*Pseudomonas fluorescens*	99.0	+	+	+	+	+	+	+	+	-
As-Tol75	Proteobacteria	Gammaproteobacteria	Burkholderiales	Comamonadaceae	*Polaromonas*	AM039831	*Polaromonas aquatica*	98.8	+	+	+	+	+	++	+	-	-
As-Tol76	Proteobacteria	Gammaproteobacteria	Pseudomonadales	Pseudomonadaceae	*Pseudomonas*	JF694789	*Pseudomonas* sp. BLH-5B9	99.2	+	+	+	+	+	++	++	+	-
**As-Tol77**	**Proteobacteria**	**Gammaproteobacteria**	**Pseudomonadales**	**Pseudomonadaceae**	***Pseudomonas***	**D86003**	***Pseudomonas fluorescens***	**97.8**	**-**	**+**	**+**	**+**	**++**	**+++**	**+**	**+**	**-**
As-Tol78	Proteobacteria	Gammaproteobacteria	Pseudomonadales	Pseudomonadaceae	*Pseudomonas*	CP026880	*Pseudomonas* sp. LH1G9	96.9	+	+	+	+	++	++++	++	++	-
As-Tol79	Proteobacteria	Gammaproteobacteria	Pseudomonadales	Pseudomonadaceae	*Pseudomonas*	CP026880	*Pseudomonas* sp. LH1G9	98.6	-	+	+	+	+	+	+	+	-
As-Tol80	Proteobacteria	Gammaproteobacteria	Burkholderiales	Comamonadaceae	*Polaromonas*	KJ615244	uncultured bacterium	98.2	-	+	+	+	+	++	+	+	-
As-Tol81	Proteobacteria	Gammaproteobacteria	Burkholderiales	Comamonadaceae	*Acidovorax*	CP027669	*Simplicispira suum*	95.9	-	+	+	+	+	+	+	+	-
As-Tol82	Proteobacteria	Gammaproteobacteria	Enterobacterales	Aeromonadaceae	*Aeromonas*	JX872320	uncultured bacterium	98.8	-	+	+	+	++	++	++	+	-
As-Tol83	Proteobacteria	Alphaproteobacteria	Rhizobiales	Xanthobacteraceae	*Tardiphaga*	KY302111	*Tardiphaga robiniae*	96.4	+	+	-	-	+	+	-	+	-
As-Tol84	Proteobacteria	Alphaproteobacteria	Caulobacterales	Caulobacteraceae	*Brevundimonas*	EU143355	*Brevundimonas basaltis*	96.5	-	+	+	+	+	++	+	-	-
As-Tol85	Proteobacteria	Gammaproteobacteria	Pseudomonadales	Pseudomonadaceae	*Pseudomonas*	MH685564	*Pseudomonas fluorescens*	97.9	-	+	+	+	++	+++	++	+	-
As-Tol86	Proteobacteria	Gammaproteobacteria	Pseudomonadales	Pseudomonadaceae	*Pseudomonas*	JF694789	*Pseudomonas* sp. BLH-5B9	99.2	-	+	+	+	+	+	+	+	+
As-Tol87	Proteobacteria	Gammaproteobacteria	Pseudomonadales	Pseudomonadaceae	*Pseudomonas*	JX997894	*Pseudomonas* sp. X-b2	97.1	+	+	+	+	+	++	+	+	-
As-Tol88	Proteobacteria	Gammaproteobacteria	Pseudomonadales	Pseudomonadaceae	*Pseudomonas*	CP026880	*Pseudomonas* sp. LH1G9	99.0	+	+	+	++	++	+++	+++	+	-
As-Tol89	Proteobacteria	Gammaproteobacteria	Pseudomonadales	Pseudomonadaceae	*Pseudomonas*	JX997894	*Pseudomonas* sp. X-b2	97.1	+	-	+	+	++	+++	+	+	-
As-Tol90				sequencing not successful						+	+	+	++	++	++	+	-
As-Tol91	Proteobacteria	Gammaproteobacteria	Pseudomonadales	Pseudomonadaceae	*Pseudomonas*	MH685564	*Pseudomonas fluorescens*	97.9	+	+	+	+	++	++	++	++	-
As-Tol92				sequencing not successful						+	+	+	+	++	+	+	-
As-Tol93	Proteobacteria	Gammaproteobacteria	Pseudomonadales	Pseudomonadaceae	*Pseudomonas*	DQ683574	*Pseudomonas* sp. MB16	96.6	+	-	-	+	+	+	-	+	-
As-Tol94				sequencing not successful						+	+	+	+	++	+	-	-
As-Tol95	Proteobacteria	Gammaproteobacteria	Pseudomonadales	Pseudomonadaceae	*Pseudomonas*	JF694789	*Pseudomonas* sp. BLH-5B9	96.2	+	+	+	+	+	++	+	-	-
As-Tol96	Proteobacteria	Alphaproteobacteria	Caulobacterales	Caulobacteraceae	*Brevundimonas*	JN009622	*Brevundimonas* sp. GC044	95.7	+	+	+	+	+	++	+	-	-
As-Tol97	Proteobacteria	Alphaproteobacteria	Caulobacterales	Caulobacteraceae	*Brevundimonas*	AF321018	*Brevundimonas* sp. GOBB3-212	94.1	+	+	+	+	+	++	+	-	-
As-Tol98				sequencing not successful						-	-	+	+	++	+	-	-
As-Tol99	Proteobacteria	Gammaproteobacteria	Burkholderiales	Comamonadaceae	*Acidovorax*	CP027669	*Simplicispira suum*	97.3	+	+	+	+	+	++	+	-	-
As-Tol100	Proteobacteria	Alphaproteobacteria	Caulobacterales	Caulobacteraceae	*Brevundimonas*	HF570063	*Brevundimonas intermedia*	94.9	+	+	+	+	+	++	+	++	+
As-Tol101	Proteobacteria	Alphaproteobacteria	Caulobacterales	Caulobacteraceae	*Brevundimonas*	AF321018	*Brevundimonas* sp. GOBB3-212	96.2	+	-	+	+	+	++	+	++	+
As-Tol102				sequencing not successful						+	+	+	+	+	+	+	+
As-Tol103				sequencing not successful						+	-	+	+	++	+	+	-
As-Tol104	Proteobacteria	Gammaproteobacteria	Burkholderiales	Comamonadaceae	*Polaromonas*	GQ254296	uncultured *Polaromonas* sp.	89.2	-	+	+	+	+	+	-	-	-
As-Tol105	Proteobacteria	Gammaproteobacteria	Burkholderiales	Comamonadaceae	*Acidovorax*	GU385755	uncultured bacterium	96.6	-	+	+	+	+	++	+	-	+
As-Tol106	Proteobacteria	Gammaproteobacteria	Burkholderiales	Comamonadaceae	*Polaromonas*	KJ615244	uncultured bacterium	98.2	-	+	+	+	+	++	+	-	-
As-Tol107	Proteobacteria	Gammaproteobacteria	Burkholderiales	Comamonadaceae	*Polaromonas*	KJ615244	uncultured bacterium	98.2	-	+	+	+	+	+	+	+	-
As-Tol108	Proteobacteria	Gammaproteobacteria	Burkholderiales	Comamonadaceae	*Polaromonas*	KJ615244	uncultured bacterium	96.5	-	+	+	+	+	+	+	-	+
As-Tol109	Proteobacteria	Gammaproteobacteria	Burkholderiales	Comamonadaceae	*Polaromonas*	KJ615244	uncultured bacterium	98.2	-	+	+	+	+	++	+	-	+
As-Tol110	Proteobacteria	Gammaproteobacteria	Burkholderiales	Comamonadaceae	*Polaromonas*	GQ254296	uncultured *Polaromonas* sp.	97.8	-	+	+	+	+	+	+	-	-
As-Tol111	Proteobacteria	Gammaproteobacteria	Burkholderiales	Comamonadaceae	*Polaromonas*	KJ615244	uncultured bacterium	96.7	-	+	+	+	+	++	+	-	+
As-Tol112	Proteobacteria	Gammaproteobacteria	Pseudomonadales	Pseudomonadaceae	*Pseudomonas*	AF184219	*Pseudomonas* sp. Ant9	97.1	-	+	+	+	+	++	+	+	-
As-Tol113	Proteobacteria	Gammaproteobacteria	Burkholderiales	Comamonadaceae	*Polaromonas*	KJ615244	uncultured bacterium	98.2	-	+	+	+	+	+	+	+	-
As-Tol114	Proteobacteria	Alphaproteobacteria	Sphingomonadales	Sphingomonadaceae	*Sphingomonas*	JQ977093	*Sphingomonas* sp. Cra4	99.6	-	+	+	+	+	++	+	-	+
As-Tol115	Proteobacteria	Gammaproteobacteria	Pseudomonadales	Pseudomonadaceae	*Pseudomonas*	AF184219	*Pseudomonas* sp. Ant9	97.8	+	+	+	+	+	+++	+	+	-
As-Tol116	Proteobacteria	Gammaproteobacteria	Pseudomonadales	Pseudomonadaceae	*Pseudomonas*	AF184219	*Pseudomonas* sp. Ant9	99.3	+	+	+	+	+	++	+	-	-
As-Tol117	Proteobacteria	Gammaproteobacteria	Pseudomonadales	Pseudomonadaceae	*Pseudomonas*	AF184219	*Pseudomonas* sp. Ant9	96.9	+	+	+	+	-	-	-	++	-

[a] Sequencing trace file did not indicate the presence of more than one strains (i.e. no mixed base calls).

Table S2: Identification and characterization of arsenite-oxidizing microorganisms from peat. Enrichments were classified based on the sequence of the dominant strain using the SILVA Alignment, Classification and Tree Service and most closely related 16S rRNA gene reference sequences were identified using BLAST. Enrichments which were further purified to obtain pure culture strains are marked in bold. Activity at different temperatures is given in categories relative to the highest observed arsenite turnover rate: (-) no observed activity (0-2% of maximum), (+) low activity (2-24% of maximum), (++) substantial activity (25-49% of maximum), (+++) high activity (50-74% of maximum), and (++++) maximum activity (75-100% of maximum).

Enrichment name	Sequencing results								Potential pure culture [a]	Growth observed at							
	SILVA classification					Next related sequence											
	Phylum	Class	Order	Family	Genus	Accession no.	Name	% similarity		2 ºC	5 ºC	10 ºC	15 ºC	20 ºC	28 ºC	33 ºC	40 ºC
As-ox-1				sequencing not successful						+	+	+	+	++	+++	+	-
As-ox-2	Proteobacteria	Alphaproteobacteria	Rhizobiales	Xanthobacteraceae	*Ancylobacter*	EU589386	*Ancylobacter dichloromethanicus*	99.4	-	+	+	+	+	+	++	++	-
As-ox-3				sequencing not successful						+	+	+	++	+	++	++	-
As-ox-4				sequencing not successful						+	+	+	+	+	+++	+	-
As-ox-5	Proteobacteria	Gammaproteobacteria	Burkholderiales	Comamonadaceae	*Rhodoferax*	JX224615	uncultured bacterium	99.8	-	+	+	+	+	+	+	+	-
**As-ox-6**				**sequencing not successful**						**+**	**+**	**+**	**+**	**+**	**++**	**+**	**-**
As-ox-7	Proteobacteria	Alphaproteobacteria	Rhizobiales	Xanthobacteraceae	*Ancylobacter*	AY056830	*Ancylobacter rudongensis*	99.8	-	+	+	+	+	++	+++	+	-
As-ox-8				sequencing not successful						+	+	+	+	++	++	+	-
As-ox-9	Proteobacteria	Gammaproteobacteria	Pseudomonadales	Pseudomonadaceae	*Pseudomonas*	CP026880	*Pseudomonas* sp. LH1G9	99.9	+	+	+	+	+	+	+++	+	-
As-ox-10				sequencing not successful						+	+	+	+	+	+	+	-
**As-ox-11**	**Proteobacteria**	**Alphaproteobacteria**	**Rhizobiales**	**Xanthobacteraceae**	***Ancylobacter***	**AY056830**	***Ancylobacter rudongensis***	**92.1**	**+**	**+**	**+**	**+**	**+**	**+**	**+++**	**+**	**-**
As-ox-12	Proteobacteria	Gammaproteobacteria	Pseudomonadales	Pseudomonadaceae	*Pseudomonas*	CP026880	*Pseudomonas* sp. LH1G9	99.9	+	+	+	+	+	+	++	++	-
As-ox-13				sequencing not successful						+	+	+	++	++	++	+	-
As-ox-14				sequencing not successful						+	+	+	+	++	+++	+	-
As-ox-15				sequencing not successful						+	+	+	+	+	+	+	-
As-ox-16	Proteobacteria	Gammaproteobacteria	Burkholderiales	Comamonadaceae	*Polaromonas*	KJ615244	uncultured bacterium	98.3	-	+	-	+	+	+	+	+	-
As-ox-17	Proteobacteria	Gammaproteobacteria	Burkholderiales	Comamonadaceae	*Polaromonas*	KJ615244	uncultured bacterium	98.9	-	+	+	+	+	+	+	+	-
As-ox-18				sequencing not successful						+	+	+	+	++	++	++	-
**As-ox-19**				**sequencing not successful**						**+**	**+**	**+**	**+**	**++**	**++**	**++**	**-**
As-ox-20				sequencing not successful						+	+	+	++	++	+++	++	-
As-ox-21	Proteobacteria	Gammaproteobacteria	Pseudomonadales	Pseudomonadaceae	*Pseudomonas*	CP026880	*Pseudomonas* sp. LH1G9	99.9	+	+	+	++	+	++	+++	+	-
As-ox-22	Proteobacteria	Alphaproteobacteria	Rhizobiales	Rhizobiaceae	*Ensifer*	MT941017	*Ensifer adhaerens* strain L1/CT4	98.9	-	+	+	+	+	+	+	+	-
As-ox-23				sequencing not successful						+	+	+	+	+	+	+	-
As-ox-24	Proteobacteria	Gammaproteobacteria	Pseudomonadales	Pseudomonadaceae	*Pseudomonas*	CP026880	*Pseudomonas* sp. LH1G9	99.9	+	+	+	+	+	++	+++	++	-
As-ox-25				sequencing not successful						+	+	+	++	++	++++	+	-
As-ox-26	Proteobacteria	Gammaproteobacteria	Pseudomonadales	Pseudomonadaceae	*Pseudomonas*	CP026880	*Pseudomonas* sp. LH1G9	99.9	+	+	+	-	-	-	-	+	-
As-ox-27				sequencing not successful						+	+	+	++	++	+++	+	-
**As-ox-28**	**Actinobacteriota**	**Actinobacteria**	**Corynebacteriales**	**Nocardiaceae**	***Rhodococcus***	**JX105591**	**uncultured bacterium**	**96.0**	**-**	**-**	**-**	**+**	**+**	**+**	**+**	**+**	**-**
As-ox-29	Proteobacteria	Alphaproteobacteria	Rhizobiales	Rhizobiaceae	*Allorhizobium-Neorhizobium-Pararhizobium-Rhizobium*	JQ764998	*Agrobacterium* sp. BE516	99.4	+	+	+	+	+	+	++	++	-
As-ox-30	Proteobacteria	Gammaproteobacteria	Pseudomonadales	Pseudomonadaceae	*Pseudomonas*	CP026880	*Pseudomonas* sp. LH1G9	99.9	+	+	+	+	+	+	++	+	-
As-ox-31				sequencing not successful						+	+	+	+	+	+++	+	-
As-ox-32	Proteobacteria	Gammaproteobacteria	Burkholderiales	Comamonadaceae	*Polaromonas*	KJ615244	uncultured bacterium	99.6	+	+	+	-	-	+	-	+	-
As-ox-33	Proteobacteria	Gammaproteobacteria	Pseudomonadales	Pseudomonadaceae	*Pseudomonas*	MH998405	*Pseudomonas* sp.	95.2	+	+	-	-	+	+	-	+	-
As-ox-34				sequencing not successful						+	+	+	+	++	+++	++	-
As-ox-35	Proteobacteria	Gammaproteobacteria	Pseudomonadales	Pseudomonadaceae	*Pseudomonas*	CP026880	*Pseudomonas* sp. LH1G9	99.9	-	+	+	+	++	++	+++	++	-
**As-ox-36**				**sequencing not successful**						**+**	**+**	**-**	**-**	**+**	**+**	**+**	**-**
As-ox-37	Proteobacteria	Alphaproteobacteria	Rhizobiales	Xanthobacteraceae	*Ancylobacter*	AY056830	*Ancylobacter rudongensis*	99.8	+	+	+	+	+	++	++	++	-
As-ox-38	Proteobacteria	Gammaproteobacteria	Pseudomonadales	Pseudomonadaceae	*Pseudomonas*	CP026880	*Pseudomonas* sp. LH1G9	99.9	-	+	+	+	+	++	++	++	-
As-ox-39	Proteobacteria	Gammaproteobacteria	Pseudomonadales	Pseudomonadaceae	*Pseudomonas*	CP026880	*Pseudomonas* sp. LH1G9	99.9	-	+	+	+	+	+	++	+	-
As-ox-40	Proteobacteria	Alphaproteobacteria	Caulobacterales	Caulobacteraceae	*Brevundimonas*	JN009622	*Brevundimonas* sp. GC044	98.7	-	-	-	+	+	+	-	+	-

[a] Sequencing trace file did not indicate the presence of more than one strains (i.e. no mixed base calls).

Table S3: Identification and characterization of arsenate-respiring microorganisms from peat. Enrichments were classified based on the sequence of the dominant strain using the SILVA Alignment, Classification and Tree Service and most closely related 16S rRNA gene reference sequences were identified using BLAST. Enrichments which were further purified to obtain pure culture strains are marked in bold. Activity at different temperatures is given in categories relative to the highest observed arsenate turnover rate: (-) no observed activity (0-2% of maximum), (+) low activity (2-24% of maximum), (++) substantial activity (25-49% of maximum), (+++) high activity (50-74% of maximum), and (++++) maximum activity (75-100% of maximum).

Enrichment name	Sequencing results									Arsenate reduction activity observed at					
	SILVA classification					Next related sequence			Potential pure culture [a]						
	Phylum	Class	Order	Family	Genus	Accession no.	Name	% similarity		5 ºC	10 ºC	20 ºC	28 ºC	33 ºC	40 ºC
As-Red-1	Proteobacteria	Gammaproteobacteria	Burkholderiales	Comamonadaceae	*Alicycliphilus*	CP051298	*Alicycliphilus denitrificans*	98.8	-	+	+	+	+	-	-
**As-Red-2**	**Proteobacteria**	**Gammaproteobacteria**	**Burkholderiales**	**Comamonadaceae**	***Rhodoferax***	**JF694806**	**bacterium BLH-8E5**	**99.2**	**-**	**-**	**+**	**+**	**+**	**-**	**-**
As-Red-3				sequencing not successful						+	+	+	+	++	-
As-Red-4				sequencing not successful						+	++	+	+	++	-
As-Red-5				sequencing not successful						-	+	+	+	++	-
As-Red-6				sequencing not successful						-	++	++	+	++	-
As-Red-7	Proteobacteria	Gammaproteobacteria	Burkholderiales	Comamonadaceae	*Polaromonas*	GQ254296	uncultured *Polaromonas* sp.	93.4	+	+	+	++	+	+	-
**As-Red-8**				**sequencing not successful**						**+**	**+**	**++**	**++**	**-**	**-**
As-Red-9				sequencing not successful						+	++	+	+	-	-
As-Red-10	Proteobacteria	Gammaproteobacteria	Burkholderiales	Comamonadaceae	*Rhodoferax*	JF694806	bacterium BLH-8E5	99.1	-	-	+	++	+	-	-
As-Red-11				sequencing not successful						+	+	+	-	-	-
As-Red-12				sequencing not successful						-	+	+	+	+	-
As-Red-13	Proteobacteria	Gammaproteobacteria	Burkholderiales	Comamonadaceae	*Rhodoferax*	JF694806	bacterium BLH-8E5	96.6	+	+	-	+	++	++	-
As-Red-14				sequencing not successful						+	+	++	+	-	-
As-Red-15	Proteobacteria	Gammaproteobacteria	Burkholderiales	Comamonadaceae	*Rhodoferax*	JF694806	bacterium BLH-8E5	96.6	+	+	+	+	-	-	-
As-Red-16	Proteobacteria	Gammaproteobacteria	Burkholderiales	Comamonadaceae	*Rhodoferax*	JF694806	bacterium BLH-8E5	99.2	-	+	++	+	+	-	-
As-Red-17				sequencing not successful						+	++	+	+	-	-
As-Red-18				sequencing not successful						+	++	-	+	+	-
As-Red-19	Proteobacteria	Gammaproteobacteria	Burkholderiales	Comamonadaceae	*Rhodoferax*	JF694806	bacterium BLH-8E5	85.7	-	+	+	+	-	-	-
As-Red-20	Proteobacteria	Gammaproteobacteria	Burkholderiales	Comamonadaceae	*Rhodoferax*	JF694806	bacterium BLH-8E5	87.6	+	+	+	+	+	-	-
As-Red-21				sequencing not successful						-	+	++	+	-	-
As-Red-22				sequencing not successful						-	++	+	+	-	-
As-Red-23				sequencing not successful						+	++	+	+	+	-
As-Red-24	Proteobacteria	Gammaproteobacteria	Burkholderiales	Comamonadaceae	*Rhodoferax*	JF694806	bacterium BLH-8E5	98.2	+	+	+	+	+	+	-
As-Red-25				sequencing not successful						+	+	+	+	-	-
As-Red-26				sequencing not successful						-	++	++	+	-	-
As-Red-27	Proteobacteria	Alphaproteobacteria	Caulobacterales	Caulobacteraceae	*Brevundimonas*	JN030509	uncultured bacterium	98.8	+	+	+	++++	++	-	-
As-Red-28				sequencing not successful						+	+	+	+	-	-
As-Red-29				sequencing not successful						+	+	+	-	+	-
As-Red-30				sequencing not successful						-	+	++	+	+	-
As-Red-31				sequencing not successful						-	+	++	+	+	-
As-Red-32				sequencing not successful						-	+	++	++	+	-
As-Red-33	Proteobacteria	Gammaproteobacteria	Pseudomonadales	Pseudomonadaceae	*Pseudomonas*	HQ202834	*Pseudomonas mandelii*	94.9	+	-	+	+	+	+	-
As-Red-34	Proteobacteria	Gammaproteobacteria	Burkholderiales	Comamonadaceae	*Rhodoferax*	JF694806	bacterium BLH-8E5	99.1	-	-	++	+	+++	+	-
As-Red-35	Proteobacteria	Gammaproteobacteria	Burkholderiales	Comamonadaceae	*Rhodoferax*	JF694806	bacterium BLH-8E5	99.1	-	-	-	+	-	-	-
As-Red-36	Proteobacteria	Gammaproteobacteria	Burkholderiales	Comamonadaceae	*Rhodoferax*	JF694806	bacterium BLH-8E5	99.1	-	-	++	++	+	-	-
As-Red-37	Proteobacteria	Gammaproteobacteria	Burkholderiales	Comamonadaceae	*Rhodoferax*	JF694806	bacterium BLH-8E5	99.2	+	+	++	+	+	+	-
As-Red-38	Proteobacteria	Gammaproteobacteria	Burkholderiales	Comamonadaceae	*Rhodoferax*	JF694806	bacterium BLH-8E5	98.0	-	-	++	+	-	+	-
As-Red-39	Proteobacteria	Gammaproteobacteria	Burkholderiales	Comamonadaceae	*Rhodoferax*	JF694806	bacterium BLH-8E5	97.3	-	-	++	-	++	+	-
As-Red-40	Proteobacteria	Gammaproteobacteria	Burkholderiales	Comamonadaceae	*Rhodoferax*	JF694806	bacterium BLH-8E5	99.2	+	+	++	+	+	+	-
As-Red-41	Proteobacteria	Gammaproteobacteria	Burkholderiales	Comamonadaceae	*Rhodoferax*	JF694806	bacterium BLH-8E5	98.4	+	-	++	+	+	-	-
As-Red-42	Proteobacteria	Gammaproteobacteria	Pseudomonadales	Pseudomonadaceae	*Pseudomonas*	CP012680	*Pseudomonas brassicacearum*	95.8	+	-	-	-	-	-	-
As-Red-43	Proteobacteria	Gammaproteobacteria	Burkholderiales	Comamonadaceae	*Rhodoferax*	JF694806	bacterium BLH-8E5	99.1	-	-	++	+	+	+	-
As-Red-44	Proteobacteria	Gammaproteobacteria	Burkholderiales	Comamonadaceae	*Rhodoferax*	JF694806	bacterium BLH-8E5	99.1	+	+	++	++	+	-	-
As-Red-45	Proteobacteria	Gammaproteobacteria	Burkholderiales	Comamonadaceae	*Rhodoferax*	JF694806	bacterium BLH-8E5	96.6	+	-	+	+	-	+	-
As-Red-46	Proteobacteria	Gammaproteobacteria	Burkholderiales	Comamonadaceae	*Rhodoferax*	JF694806	bacterium BLH-8E5	99.1	-	+	++	+	+	-	-
As-Red-47	Proteobacteria	Gammaproteobacteria	Burkholderiales	Comamonadaceae	*Rhodoferax*	JF694806	bacterium BLH-8E5	97.3	-	-	++	-	++	+	-
As-Red-48	Proteobacteria	Gammaproteobacteria	Burkholderiales	Comamonadaceae	*Rhodoferax*	JF694806	bacterium BLH-8E5	97.3	+	-	++	+	+	-	-
As-Red-49	Proteobacteria	Gammaproteobacteria	Burkholderiales	Comamonadaceae	*Rhodoferax*	JF694806	bacterium BLH-8E5	97.3	+	-	-	++	++	+	-
As-Red-50	Proteobacteria	Gammaproteobacteria	Burkholderiales	Comamonadaceae	*Rhodoferax*	JF694806	bacterium BLH-8E5	99.1	-	-	+	+	+	-	-
As-Red-51	Proteobacteria	Gammaproteobacteria	Burkholderiales	Comamonadaceae	*Rhodoferax*	JF694806	bacterium BLH-8E5	99.1	-	-	++	+	+	-	-
As-Red-52	Proteobacteria	Gammaproteobacteria	Burkholderiales	Comamonadaceae	*Rhodoferax*	JF694806	bacterium BLH-8E5	99.1	+	-	+	++	++	-	-
As-Red-53	Proteobacteria	Gammaproteobacteria	Burkholderiales	Comamonadaceae	*Rhodoferax*	JF694806	bacterium BLH-8E5	97.3	+	-	+	+	+	-	-
As-Red-54	Proteobacteria	Gammaproteobacteria	Burkholderiales	Comamonadaceae	*Rhodoferax*	JF694806	bacterium BLH-8E5	97.7	-	+	++	+	++	+	-
As-Red-55	Proteobacteria	Gammaproteobacteria	Burkholderiales	Comamonadaceae	*Rhodoferax*	JF694806	bacterium BLH-8E5	95.8	-	+	++	+	-	-	-
As-Red-56	Proteobacteria	Gammaproteobacteria	Burkholderiales	Comamonadaceae	*Rhodoferax*	JF694806	bacterium BLH-8E5	99.1	-	-	-	+	-	+	-
As-Red-57	Proteobacteria	Gammaproteobacteria	Burkholderiales	Comamonadaceae	*Rhodoferax*	JF694806	bacterium BLH-8E5	98.4	+	-	++	+	-	-	-
As-Red-58				sequencing not successful						-	-	+	++++	+	-
As-Red-59	Proteobacteria	Gammaproteobacteria	Burkholderiales	Comamonadaceae	*Rhodoferax*	JF694806	bacterium BLH-8E5	97.3	+	-	++	-	+	+	-
As-Red-60	Proteobacteria	Gammaproteobacteria	Burkholderiales	Comamonadaceae	*Rhodoferax*	JF694806	bacterium BLH-8E5	97.8	-	-	-	-	-	-	-
As-Red-61	Proteobacteria	Gammaproteobacteria	Burkholderiales	Comamonadaceae	*Rhodoferax*	JF694806	bacterium BLH-8E5	99.1	-	-	+	+	++	+	-
As-Red-62	Proteobacteria	Gammaproteobacteria	Burkholderiales	Comamonadaceae	*Rhodoferax*	JF694806	bacterium BLH-8E5	97.6	+	-	++	-	+	-	-
As-Red-63	Proteobacteria	Gammaproteobacteria	Burkholderiales	Comamonadaceae	*Rhodoferax*	JF694806	bacterium BLH-8E5	99.2	-	-	++	+	-	-	-
As-Red-64				sequencing not successful						-	+	++	+++	+	-
As-Red-65	Proteobacteria	Gammaproteobacteria	Enterobacterales	Yersiniaceae	*Rahnella*	CP032296	*Rahnella aquatilis*	98.4	-	-	++	++	+	+	-
As-Red-66	Proteobacteria	Gammaproteobacteria	Burkholderiales	Comamonadaceae	*Rhodoferax*	JF694806	bacterium BLH-8E5	99.1	+	-	++	+	+++	++	-
As-Red-67	Proteobacteria	Gammaproteobacteria	Burkholderiales	Comamonadaceae	*Rhodoferax*	JF694806	bacterium BLH-8E5	99.1	-	-	+	+	+	-	-
**As-Red-68**	**Proteobacteria**	**Gammaproteobacteria**	**Pseudomonadales**	**Pseudomonadaceae**	***Pseudomonas***	**CP005960**	***Pseudomonas mandelii* JR-1**	**99.5**	**+**	**-**	**++**	**+**	**+++**	**+**	**-**
As-Red-69	Proteobacteria	Gammaproteobacteria	Burkholderiales	Comamonadaceae	*Rhodoferax*	JF694806	bacterium BLH-8E5	98.2	-	-	++	++	+	-	-
As-Red-70	Proteobacteria	Gammaproteobacteria	Burkholderiales	Comamonadaceae	*Rhodoferax*	JF694806	bacterium BLH-8E5	99.1	-	-	++	-	-	-	-
As-Red-71				sequencing not successful						-	+	+	-	-	-
As-Red-72	Proteobacteria	Gammaproteobacteria	Burkholderiales	Oxalobacteraceae	*Duganella*	MT380163	*Duganella zoogloeoides*	81.6	-	-	+	-	-	+	-
As-Red-73	Proteobacteria	Gammaproteobacteria	Burkholderiales	Comamonadaceae	*Limnohabitans*	JX521359	uncultured bacterium	95.6	+	-	++	++	++	+	-
As-Red-74	Proteobacteria	Gammaproteobacteria	Burkholderiales	Comamonadaceae	*Rhodoferax*	JF694806	bacterium BLH-8E5	96.6	-	-	++	++	+++	+	-
As-Red-75	Proteobacteria	Gammaproteobacteria	Burkholderiales	Comamonadaceae	*Rhodoferax*	JF694806	bacterium BLH-8E5	99.2	+	-	++	++	++	+	-
As-Red-76				sequencing not successful						-	++	+++	++++	+	-

[a] Sequencing trace file did not indicate the presence of more than one strains (i.e. no mixed base calls).

Table S4: Identification and characterization of arsenic metabolizers from peat. Isolates were classified using the SILVA Alignment, Classification and Tree Service or PROTAX-fungi and most closely related 16S rRNA gene or ITS reference sequences were identified using BLAST.

	Isolate name	Obtained from enrichment	Sequencing results									Comparison to metagenome			
			SILVA/PROTAX-fungi classification						Next related sequence			Matches to metagenome sequences (100% identity)		Annotated reads in metagenome on genus level (%)	
			Kingdom	Phylum	Class	Order	Family	Genus	Accession no.	Name	% similarity	Number of matches	Average (min-max) sequence length (bp)	RefSeq	SILVA SSU
Arsenic-tolerant microorganisms	As-Tol-iso-1	As-Tol2	Bacteria	Proteobacteria	Gammaproteobacteria	Pseudomonadales	Pseudomonadaceae	*Pseudomonas*	MG576154	*Pseudomonas rhodesiae*	99.5	199	41 (28-151)	0.78	0.13
	As-Tol-iso-2	As-Tol69	Bacteria	Actinobacteriota	Actinobacteria	Corynebacteriales	Nocardiaceae	*Rhodococcus*	KR085922	*Rhodococcus yunnanensis*	99.9	121	60 (28-151)	0.25	0.26
	As-Tol-iso-3	As-Tol41	Bacteria	Proteobacteria	Gammaproteobacteria	Pseudomonadales	Pseudomonadaceae	*Pseudomonas*	KX549994	*Pseudomonas azotoformans*	99.7	168	40 (28-151)	0.78	0.13
	As-Tol-iso-4	As-Tol53	Bacteria	Proteobacteria	Gammaproteobacteria	Pseudomonadales	Pseudomonadaceae	*Pseudomonas*	EU169178	*Pseudomonas tolaasii*	99.8	168	40 (28-151)	0.78	0.13
	As-Tol-iso-5	As-Tol70	Bacteria	Proteobacteria	Gammaproteobacteria	Pseudomonadales	Pseudomonadaceae	*Pseudomonas*	MN449462	*Pseudomonas veronii*	99.7	183	41 (28-151)	0.78	0.13
	As-Tol-iso-6	As-Tol61	Bacteria	Proteobacteria	Gammaproteobacteria	Pseudomonadales	Pseudomonadaceae	*Pseudomonas*	CP026880	*Pseudomonas* sp. LH1G9	99.6	168	42 (28-151)	0.78	0.13
	As-Tol-iso-7	As-Tol72	Bacteria	Proteobacteria	Gammaproteobacteria	Pseudomonadales	Pseudomonadaceae	*Pseudomonas*	MN449462	*Pseudomonas veronii*	99.5	184	41 (28-151)	0.78	0.13
	As-Tol-iso-8	As-Tol77	Bacteria	Proteobacteria	Gammaproteobacteria	Pseudomonadales	Pseudomonadaceae	*Pseudomonas*	DQ178231	*Pseudomonas fluorescens*	99.7	177	41 (28-151)	0.78	0.13
Arsenite-oxidizing microorganisms	As-ox-iso-1	As-ox-11	Bacteria	Actinobacteriota	Actinobacteria	Micrococcales	Microbacteriaceae	*Microbacterium*	MW391653	*Microbacterium oxydans*	100	79	41 (28-73)	0	0.05
	As-ox-iso-2	As-ox-28	Bacteria	Actinobacteriota	Actinobacteria	Corynebacteriales	Nocardiaceae	*Rhodococcus*	KM507709	*Rhodococcus* sp. FXJ8.138	99.3	119	54 (28-151)	0.25	0.26
	As-ox-iso-3	As-ox-19	Bacteria	Actinobacteriota	Actinobacteria	Corynebacteriales	Nocardiaceae	*Rhodococcus*	KM507709	*Rhodococcus* sp. FXJ8.138	99.6	138	54 (28-151)	0.25	0.26
	As-ox-iso-4	As-ox-6	Bacteria	Actinobacteriota	Actinobacteria	Micrococcales	Microbacteriaceae	*Microbacterium*	MW433631	*Microbacterium maritypicum*	100	79	41 (28-73)	0	0.05
	As-ox-iso-5	As-ox-36	Fungi	Ascomycota	Leotiomycetes	Helotiales		*Cadophora*	MK163748	*Cadophora* sp. Isolate 414 02-D	99.2	30	44 (36-53)	0	0
Arsenate-respiring microorganisms	As-Red-iso-1	As-red-2	Bacteria	Proteobacteria	Gammaproteobacteria	Pseudomonadales	Pseudomonadaceae	*Pseudomonas*	KU507610	*Pseudomonas* sp. w14-9	99.6	146	41 (28-151)	0.78	0.13
	As-Red-iso-2	As-red-68	Bacteria	Proteobacteria	Gammaproteobacteria	Pseudomonadales	Pseudomonadaceae	*Pseudomonas*	KU507610	*Pseudomonas* sp. w14-9	99.7	69	43 (28-151)	0.78	0.13
	As-Red-iso-3	As-red-8	Bacteria	Proteobacteria	Gammaproteobacteria	Pseudomonadales	Pseudomonadaceae	*Pseudomonas*	CP026880	*Pseudomonas* sp. LH1G9	99.9	168	42 (28-151)	0.78	0.13

Table S5: Detected *arsC* in the peat metagenome. Sequences of *arsC* were detected by searching the metagenome against a custom database containing reference sequences and *arsC obtained in an earlier amplicon-based study from the same site* using Blast+. Only matches with a minimum length of 100 bp were taken into account.

No. of matching sequences	Metagenome matches								
	NCBI Taxonomy						Next related sequence		
	Kingdom	Phylum	Class	Order	Family	Genus	Accession no.	Name	% similarity
1	Archaea	Euryarchaeota	Methanomicrobia	Methanosarcinales	Candidatus Methanoperedenaceae	*Candidatus Methanoperedens*	KPQ43284	Cand. *Methanoperedens* sp. BLZ1	88.6
1	Archaea	Euryarchaeota	Methanomicrobia	Methanosarcinales	Candidatus Methanoperedenaceae	*Candidatus Methanoperedens*	WP_097300620	Cand. *Methanoperedens* sp. BLZ1	91.8
1	Archaea		Cand. Methanofastidiosa			Cand. *Methanofastidiosum*	KYC44743	Cand. *Methanofastidiosum methylthiophilus*	67.4
2	Bacteria	Acidobacteria	Acidobacteriia	Acidobacteriales	Acidobacteriaceae	Cand. *Sulfotelmatomonas*	WP_207766770	Cand. *Sulfotelmatomonas gaucii*	82.0 - 90.0
1	Bacteria	Actinobacteria	Acidimicrobiia	Acidimicrobiales	Microthrixaceae	Cand. *Microthrix*	MBK6311664	Cand. *Microthrix sp.*	94.1
1	Bacteria	Actinobacteria	Actinomycetia	Catenulisporales	Catenulisporaceae	*Catenulispora*	OLE20109	*Catenulispora* sp.	85.4
1	Bacteria	Actinobacteria	Actinomycetia	Glycomycetales	Glycomycetaceae	*Stackebrandtia*	WP_142044062	*Stackebrandtia endophytica*	97.4
1	Bacteria	Actinobacteria	Actinomycetia	Micrococcales	Microbacteriaceae	*Microbacterium*	WP_193596890	*Microbacterium* sp. YJN-G	96.9
1	Bacteria	Actinobacteria	Actinomycetia	Streptomycetales	Streptomycetaceae	*Streptomyces*	WP_030673875	*Streptomyces cellulosae*	90.9
1	Bacteria	Actinobacteria	Rubrobacteria	Rubrobacterales	Rubrobacteraceae	*Rubrobacter*	WP_011565798	*Rubrobacter xylanophilus*	63.9
1	Bacteria	Bacteroidetes	Flavobacteriia	Flavobacteriales	Flavobacteriaceae	*Flavobacterium*	WP_114676466	*Flavobacterium arcticum*	98.0
4	Bacteria	Calditrichaeota	Calditrichae	Calditrichales	Calditrichaceae	*Caldithrix*	WP_006929893	*Caldithrix abyssi*	73.6-78.0
3	Bacteria	Cand. Omnitrophica				Cand. *Vampirococcus*	WP_128700398	Cand. *Vampirococcus archaeovorus*	85.7 - 87.8
2	Bacteria	Cand. Hydrogenedentes				Cand. *Hydrogenedens*	NLF56712	Cand. *Hydrogenedens* sp.	80.0 - 84.8
1	Bacteria	Cand. Kryptonia				Cand. *Kryptobacter*	CUS96286	Cand. *Kryptobacter tengchongensis*	71.4
1	Bacteria	Cand. Kryptonia				Cand. *Kryptonium*	CUS77150	Cand. *Kryptonium thompsoni*	66.7
1	Bacteria	Chlorobi	Chlorobia	Chlorobiales	Chlorobiaceae	*Chlorobium*	MBL6956640	*Chlorobium phaeobacteroides*	66.7
1	Bacteria	Chlorobi	Chlorobia	Chlorobiales	Chlorobiaceae	*Prosthecochloris*	WP_094082804	*Prosthecochloris* sp. GSB1	80.5
10	Bacteria	Chloroflexi	Anaerolineae	Aggregatilineales	Aggregatilineaceae	*Aggregatilinea*	WP_119069892	*Aggregatilinea lenta*	63.8-73.5
13	Bacteria	Chloroflexi	Anaerolineae	Anaerolineales	Anaerolineaceae	*Anaerolinea*	HGS22719	*Anaerolinea thermolimosa*	65.3 - 78.4
2	Bacteria	Chloroflexi	Anaerolineae	Anaerolineales	Anaerolineaceae	*Anaerolinea*	WP_084001499	*Anaerolinea thermolimosa*	64.6 - 79.5
1	Bacteria	Chloroflexi	Anaerolineae	Anaerolineales	Anaerolineaceae	*Bellilinea*	WP_061919389	*Bellilinea caldifistulae*	72.2
4	Bacteria	Chloroflexi	Anaerolineae	Anaerolineales	Anaerolineaceae	*Levilinea*	WP_062417446	*Levilinea saccharolytica*	66.0 - 73.1
10	Bacteria	Chloroflexi	Anaerolineae	Anaerolineales	Anaerolineaceae	*Ornatilinea*	WP_075061747	*Ornatilinea apprima*	66.0 - 73.2
4	Bacteria	Chloroflexi	Ardenticatenia	Ardenticatenales	Ardenticatenaceae	*Candidatus Promineofilum*	MBP6787624	Cand. *Promineofilum* sp.	67.4 - 72.3
3	Bacteria	Chloroflexi	Caldilineae	Caldilineales	Caldilineaceae	*Caldilinea*	WP_014434771	*Caldilinea aerophila*	68.1 - 82.4
3	Bacteria	Chloroflexi	Candidatus Thermofonsia			*Candidatus Roseilinea*	WP_169237713	Cand. *Roseilinea* sp. NK_OTU-006	65.3 - 71.1
2	Bacteria	Chloroflexi	Thermoflexia	Thermoflexales	Thermoflexaceae	*Thermoflexus*	GBD09074	Cand. *Thermoflexus japonica*	65.2 - 67.4
1	Bacteria	Chloroflexi				*Thermobaculum*	WP_012876586	*Thermobaculum terrenum*	64.6
2	Bacteria	Cyanobacteria		Synechococcales	Synechococcaceae	*Synechococcus*	WP_017325974	*Synechococcus* sp. PCC 7336	77.6 - 79.1
1	Bacteria	Deferribacteres	Deferribacteres	Deferribacterales	Deferribacteraceae	*Denitrovibrio*	WP_211204119	*Denitrovibrio acetiphilus*	73.2
1	Bacteria	Deinococcus-Thermus	Deinococci	Thermales	Thermaceae	*Meiothermus*	WP_013157596	*Meiothermus silvanus*	75.0
5	Bacteria	Fibrobacteres	Fibrobacteria	Fibrobacterales	Fibrobacteriaceae	*Fibrobacter*	NLD99685	*Fibrobacter* sp.	68.3 - 86.7
1	Bacteria	Nitrospinae	Nitrospinia	Nitrospinales	Nitrospinaceae	*Nitrospina*	HBP10378	*Nitrospina* sp.	80.0
3	Bacteria	Planctomycetes	Phycisphaerae	Sedimentisphaerales	Sedimentipshaeraceae	*Limihaloglobus*	WP_146682309	*Limihaloglobus sulfuriphilus*	71.4 - 82.6
11	Bacteria	Planctomycetes	Phycisphaerae	Sedimentisphaerales	Sedimentipshaeraceae	*Sedimentisphaera*	WP_077541149	*Sedimentisphaera cyanobacteriorum*	70.8 - 91.3
1	Bacteria	Planctomycetes	Phycisphaerae	Sedimentisphaerales	Sedimentipshaeraceae	*Sedimentisphaera*	WP_094759900	*Sedimentisphaera salicampi*	70.2
2	Bacteria	Planctomycetes	Planctomycetia	Pirellulales	Lacipirellulaceae	*Posidoniimonas*	WP_146568586	*Posidoniimonas corsicana*	73.5 - 76.0
1	Bacteria	Planctomycetes	Planctomycetia	Pirellulales	Lacipirellulaceae	*Pseudobythopirellula*	WP_146395466	*Pseudobythopirellula maris*	86.1
1	Bacteria	Planctomycetes	Planctomycetia	Pirellulales	Thermoguttaceaea	*Thermogutta*	WP_207651826	*Thermogutta terrifontis*	75.6
2	Bacteria	Planctomycetes	Planctomycetia	Planctomycetales	Planctomycetaceae	*Gimesia*	WP_145181926	*Gimesia chilikensis*	61.2 - 66.0
3	Bacteria	Planctomycetes	Planctomycetia	Planctomycetales	Planctomycetaceae	*Maioricimonas*	WP_145369353	*Maioricimonas rarisocia*	71.8 - 76.7
1	Bacteria	Planctomycetes	Planctomycetia	Planctomycetales	Planctomycetaceae	*Planctomyces*	MBB02950	*Planctomyces* sp.	80.0
2	Bacteria	Planctomycetes	Planctomycetia	Planctomycetales	Planctomycetaceae	*Rubinisphaera*	WP_013629310	*Rubinisphaera brasiliensis*	75.7
1	Bacteria	Proteobacteria	Alphaproteobacteria	Caulobacterales	Caulobacteraceae	*Caulobacter*	WP_012285834	*Caulobacter* sp. K31	88.1
1	Bacteria	Proteobacteria	Alphaproteobacteria	Caulobacterales	Caulobacteraceae	*Phenylobacterium*	MBP9232352	*Phenylobacterium* sp.	92.9
1	Bacteria	Proteobacteria	Alphaproteobacteria	Hyphomicrobiales	Ahrensiaceae	*Ahrensia*	WP_018687670	*Ahrensia kielensis*	85.0
1	Bacteria	Proteobacteria	Alphaproteobacteria	Hyphomicrobiales	Beijerinckiaceae	*Methylovirgula*	WP_116400401	*Methylovirgula* sp. 4M-Z18	82.5
1	Bacteria	Proteobacteria	Alphaproteobacteria	Hyphomicrobiales	Devosiaceae	*Devosia*	WP_067456046	*Devosia elaeis*	92.1
1	Bacteria	Proteobacteria	Alphaproteobacteria	Hyphomicrobiales	Hyphomicrobiaceae	*Rhodoplanes*	WP_068023789	*Rhodoplanes* sp. Z2-YC6860	88.2
2	Bacteria	Proteobacteria	Alphaproteobacteria	Rhodospirillales	Rhodospirillaceae	*Rhodospirillum*	WP_207187434	*Rhodospirillum rubrum*	97.7
1	Bacteria	Proteobacteria	Alphaproteobacteria			*Phreatobacter*	WP_137098051	*Phreatobacter* sp. NMCR1094	83.3
1	Bacteria	Proteobacteria	Betaproteobacteria	Burkholderiales	Alcaligenaceae	*Pigmentiphaga*	WP_087839903	*Pigmentiphaga* sp.	91.4
1	Bacteria	Proteobacteria	Betaproteobacteria	Burkholderiales	Comamonadaceae	*Oryzisolibacter*	WP_091570963	*Oryzisolibacter propanilivorax*	90.6
5	Bacteria	Proteobacteria	Betaproteobacteria	Burkholderiales		*Rubrivivax*	MBC7932451	*Rubrivivax* sp.	67.9 - 72.1
1	Bacteria	Proteobacteria	Betaproteobacteria	Nitrosomonadales	Thiobacillaceae	*Thiobacillus*	NTV97515	*Thiobacillus* sp.	94.1
1	Bacteria	Proteobacteria	Betaproteobacteria	Rhodocyclales	Zoogloeaceae	*Azoarcus*	WP_212353419	*Azoarcus* sp. L1K30	89.5
5	Bacteria	Proteobacteria	Betaproteobacteria	Rhodocyclales	Zoogloeaceae	*Zoogloea*	NTV11520	*Zoogloea* sp.	79.2 - 89.5
1	Bacteria	Proteobacteria	Deltaproteobacteria	Desulfobacterales	Desulfobacteraceae	*Desulfatirhabdium*	WP_051328248	*Desulfatirhabdium butyrativorans*	69.4
2	Bacteria	Proteobacteria	Deltaproteobacteria	Desulfobacterales	Desulfobacteraceae	*Desulfobacter*	WP_020588928	*Desulfobacter curvatus*	85.4 - 86.1
2	Bacteria	Proteobacteria	Deltaproteobacteria	Desulfobacterales	Desulfobacteraceae	*Desulfoluna*	WP_092207991	*Desulfoluna spongiiphila*	76.6 - 79.0
1	Bacteria	Proteobacteria	Deltaproteobacteria	Desulfobacterales	Desulfobacteraceae	*Desulforegula*	WP_027357711	*Desulforegula conservatrix*	87.8
1	Bacteria	Proteobacteria	Deltaproteobacteria	Desulfobacterales	Desulfobacteraceae	*Desulfosarcina*	WP_155320037	*Desulfosarcina alkanivorans*	83.9
3	Bacteria	Proteobacteria	Deltaproteobacteria	Desulfobacterales	Desulfobulbaceae	*Desulfobulbus*	HEB50521	*Desulfobulbus* sp.	79.2 - 97.1
3	Bacteria	Proteobacteria	Deltaproteobacteria	Desulfobacterales	Desulfobulbaceae	*Desulfobulbus*	WP_015723982	*Desulfobulbus propionicus*	77.5 - 85.4
1	Bacteria	Proteobacteria	Deltaproteobacteria	Desulfobacterales	Desulfobulbaceae	*Desulfoprunum*	WP_183347723	*Desulfoprunum benzoelyticum*	83.8
1	Bacteria	Proteobacteria	Deltaproteobacteria	Desulfovibrionales	Desulfohalobiaceae	*Desulfonatronovibrio*	RQD61778	*Desulfonatronovibrio* sp. MSAO_Bac4	65.3
1	Bacteria	Proteobacteria	Deltaproteobacteria	Desulfovibrionales	Desulfonatronaceae	*Desulfonatronum*	WP_045221430	*Desulfonatronum thioautotrophicum*	86.1
2	Bacteria	Proteobacteria	Deltaproteobacteria	Desulfovibrionales	Desulfonatronaceae	*Desulfonatronum*	WP_107739653.	*Desulfonatronum* sp. SC1	77.6 - 80.0
6	Bacteria	Proteobacteria	Deltaproteobacteria	Desulfovibrionales	Desulfovibrionaceae	*Desulfocurvibacter*	WP_005987549	*Desulfocurvibacter africanus*	72.0 - 83.7
3	Bacteria	Proteobacteria	Deltaproteobacteria	Desulfovibrionales	Desulfovibrionaceae	*Desulfolutivibrio*	WP_176630766	*Desulfolutivibrio sulfoxidireducens*	74.0 - 76.0
1	Bacteria	Proteobacteria	Deltaproteobacteria	Desulfovibrionales	Desulfovibrionaceae	*Desulfovibrio*	MBI4806304	*Desulfovibrio* sp.	68.1
1	Bacteria	Proteobacteria	Deltaproteobacteria	Desulfovibrionales	Desulfovibrionaceae	*Desulfovibrio*	WP_018124821	*Desulfovibrio oxyclinae*	83.3
1	Bacteria	Proteobacteria	Deltaproteobacteria	Desulfovibrionales	Desulfovibrionaceae	*Desulfovibrio*	WP_174403550	*Desulfovibrio subterraneus*	71.4
1	Bacteria	Proteobacteria	Deltaproteobacteria	Desulfuromonadales	Geobacteraceae	*Geobacter*	HBA88131	*Geobacter* sp.	76.5
1	Bacteria	Proteobacteria	Deltaproteobacteria	Desulfuromonadales	Geobacteraceae	*Geobacter*	WP_012468680	*Geobacter lovleyi*	69.4
2	Bacteria	Proteobacteria	Deltaproteobacteria	Desulfuromonadales	Geobacteraceae	*Geobacter*	WP_078790332	*Geobacter thiogenes*	71.0 - 74.5
6	Bacteria	Proteobacteria	Deltaproteobacteria	Syntrophobacterales	Syntrophaceae	*Smithella*	OPY86753	*Smithella* sp. PtaU1.Bin162	62.2 - 73.0
11	Bacteria	Proteobacteria	Deltaproteobacteria	Syntrophobacterales	Syntrophaceae	*Syntrophus*	OPY19142	*Syntrophus* sp. PtaB.Bin075	64.0 - 73.5
9	Bacteria	Proteobacteria	Deltaproteobacteria	Syntrophobacterales	Syntrophaceae	*Syntrophus*	WP_011417387	*Syntrophus aciditrophicus*	61.7 - 75.5
2	Bacteria	Proteobacteria	Deltaproteobacteria	Syntrophobacterales	Syntrophobacteraceae	*Syntrophobacter*	WP_106821703	*Syntrophobacter* sp. SbD1	66.0 - 66.7
3	Bacteria	Verrucomicrobia	Opitutae	Opitutales	Opitutaceae	*Opitutus*	MSU48050	*Opitutus* sp.	82.2 - 92.2%
5	Bacteria	Verrucomicrobia	Verrucomicrobiae	Verrucomicrobiales	Verrucomicrobia subdivision 3	*Pedosphaera*	MSR66877	*Pedosphaera* sp.	82.0 - 87.8
1	Bacteria	Verrucomicrobia	Verrucomicrobiae	Verrucomicrobiales	Verrucomicrobiaceae	*Brevifollis*	WP_146856260	*Brevifollis gellanilyticus*	87.5

Table S6: Detected *aioA* in the peat metagenome. Sequences of *aioA* were detected by searching the metagenome against a custom database containing reference sequences and *aioA* obtained in an earlier amplicon-based study from the same site using Blast+. Only matches with a minimum length of 100 bp were taken into account.

No. of matching sequences	Metagenome matches								
	NCBI Taxonomy						Next related sequence		
	Kingdom	Phylum	Class	Order	Family	Genus	Accession no.	Name	% similarity
2	Bacteria	Proteobacteria	Alphaproteobacteria	Caulobacterales	Caulobacteraceae	*Caulobacter*	WP_013079615	*Caulobacter segnis*	87.8 - 96.0
1	Bacteria	Proteobacteria	Alphaproteobacteria	Hyphomicrobiales	Alsobacteraceae	*Alsobacter*	WP_106335889	*Alsobacter soli*	86.0
1	Bacteria	Proteobacteria	Alphaproteobacteria	Hyphomicrobiales	Aurantimonadaceae	*Aurantimonas*	WP_183205811	*Aurantimonas endophytica*	77.8
1	Bacteria	Proteobacteria	Alphaproteobacteria	Hyphomicrobiales	Aurantimonadaceae	*Aureimonas*	WP_188849029	*Aureimonas glaciei*	94.5
1	Bacteria	Proteobacteria	Alphaproteobacteria	Hyphomicrobiales	Beijerinckiaceae	*Rhodoblastus*	WP_184428121	*Rhodoblastus acidophilus*	97.4
1	Bacteria	Proteobacteria	Alphaproteobacteria	Hyphomicrobiales	Blastochloridaceae	*Blastochloris*	WP_126398204	*Blastochloris tepida*	100
3	Bacteria	Proteobacteria	Alphaproteobacteria	Hyphomicrobiales	Boseaceae	*Bosea*	WP_108045171	*Bosea* sp. 124	95.9 - 100
3	Bacteria	Proteobacteria	Alphaproteobacteria	Hyphomicrobiales	Bradyrhizobiaceae	*Afipia*	NGX95150	Candidatus *Afipia apatlaquensis*	84 - 100
1	Bacteria	Proteobacteria	Alphaproteobacteria	Hyphomicrobiales	Bradyrhizobiaceae	*Bradyrhizobium*	WP_065747631	*Bradyrhizobium* sp. LMTR 3	96.0
1	Bacteria	Proteobacteria	Alphaproteobacteria	Hyphomicrobiales	Bradyrhizobiaceae	*Nitrobacter*	WP_011505135	*Nitrobacter hamburgensis*	95.1
1	Bacteria	Proteobacteria	Alphaproteobacteria	Hyphomicrobiales	Chelatococcaceae	*Chelatococcus*	WP_055460277	*Chelatococcus*	89.6
2	Bacteria	Proteobacteria	Alphaproteobacteria	Hyphomicrobiales	Devosiaceae	*Devosia*	MBN9307513	*Devosia* sp.	86.9 - 100
1	Bacteria	Proteobacteria	Alphaproteobacteria	Hyphomicrobiales	Hyphomicrobiaceae	*Aquabacter*	WP_132034721	*Aquabacter spiritensis*	91.8
2	Bacteria	Proteobacteria	Alphaproteobacteria	Hyphomicrobiales	Hyphomicrobiaceae	*Rhodomicrobium*	WP_088343886	*Rhodomicrobium*	88.4 - 92.9
2	Bacteria	Proteobacteria	Alphaproteobacteria	Hyphomicrobiales	Kaistiaceae	*Bauldia*	KAB2875637	*Bauldia* sp.	86.7 - 87.0
1	Bacteria	Proteobacteria	Alphaproteobacteria	Hyphomicrobiales	Kaistiaceae	*Kaistia*	ODT19698	*Kaistia* sp. SCN 65-12	85.1
1	Bacteria	Proteobacteria	Alphaproteobacteria	Hyphomicrobiales	Methylobacteriaceae	*Methylobacterium*	ODT45194	*Methylobacterium* sp. SCN 67-24	95.9
2	Bacteria	Proteobacteria	Alphaproteobacteria	Hyphomicrobiales	Phyllobacteriaceae	*Aminobacter*	MBB6469533	*Aminobacter lissarensis*	100
2	Bacteria	Proteobacteria	Alphaproteobacteria	Hyphomicrobiales	Phyllobacteriaceae	*Aminobacter*	WP_067963469	*Aminobacter*	100
5	Bacteria	Proteobacteria	Alphaproteobacteria	Hyphomicrobiales	Phyllobacteriaceae	*Mesorhizobium*	RWK53644	*Mesorhizobium* sp.	91.5 - 100
2	Bacteria	Proteobacteria	Alphaproteobacteria	Hyphomicrobiales	Phyllobacteriaceae	*Mesorhizobium*	WP_097572612	*Mesorhizobium sanjuanii*	93.6 - 100
1	Bacteria	Proteobacteria	Alphaproteobacteria	Hyphomicrobiales	Phyllobacteriaceae	*Mesorhizobium*	WP_106727326	*Mesorhizobium soli*	89.6
2	Bacteria	Proteobacteria	Alphaproteobacteria	Hyphomicrobiales	Phyllobacteriaceae	*Mesorhizobium*	WP_126700892	*Mesorhizobium carbonis*	100
2	Bacteria	Proteobacteria	Alphaproteobacteria	Hyphomicrobiales	Phyllobacteriaceae	*Mesorhizobium*	WP_145717069	*Mesorhizobium tianshanense*	100
1	Bacteria	Proteobacteria	Alphaproteobacteria	Hyphomicrobiales	Phyllobacteriaceae	*Mesorhizobium*	WP_187971576	*Mesorhizobium* sp. N3	100
3	Bacteria	Proteobacteria	Alphaproteobacteria	Hyphomicrobiales	Phyllobacteriaceae	*Oricola*	WP_193176966	*Oricola* sp. NBU1457	97.8 - 100
1	Bacteria	Proteobacteria	Alphaproteobacteria	Hyphomicrobiales	Phyllobacteriaceae	*Phyllobacterium*	WP_183663339	*Phyllobacterium trifolii*	100
4	Bacteria	Proteobacteria	Alphaproteobacteria	Hyphomicrobiales	Phyllobacteriaceae	*Pseudaminobacter*	WP_128627613	*Pseudaminobacter arsenicus*	90.7 - 100
1	Bacteria	Proteobacteria	Alphaproteobacteria	Hyphomicrobiales	Rhizobiaceae	*Ciceribacter*	WP_114364095	*Ciceribacter lividus*	84.6
4	Bacteria	Proteobacteria	Alphaproteobacteria	Hyphomicrobiales	Rhizobiaceae	*Ciceribacter*	WP_182305201	*Ciceribacter thiooxidans*	100
1	Bacteria	Proteobacteria	Alphaproteobacteria	Hyphomicrobiales	Rhizobiaceae	*Pseudorhizobium*	WP_052642472	*Pseudorhizobium banfieldiae*	91.8
1	Bacteria	Proteobacteria	Alphaproteobacteria	Hyphomicrobiales	Rhizobiaceae	*Rhizobium*	WP_168833882	*Rhizobium* sp. P32RR-XVIII	86.5
1	Bacteria	Proteobacteria	Alphaproteobacteria	Hyphomicrobiales	Rhizobiaceae	*Sinorhizobium*	ACS29284	*Sinorhizobium* sp. GW3	98.0
2	Bacteria	Proteobacteria	Alphaproteobacteria	Hyphomicrobiales	Roseiarcaceae	*Roseiarcus*	WP_113891880	*Roseiarcus fermentans*	86.5 - 97.9
1	Bacteria	Proteobacteria	Alphaproteobacteria	Hyphomicrobiales	Stappiaceae	*Labrenzia*	WP_116699705	*Labrenzia* sp. 011	95.9
2	Bacteria	Proteobacteria	Alphaproteobacteria	Hyphomicrobiales	Stappiaceae	*Stappia*	ORE91737	*Stappia* sp. 22II-S9-Z10	80.6 - 91.8
1	Bacteria	Proteobacteria	Alphaproteobacteria	Hyphomicrobiales	Xanthobacteraceae	*Ancylobacter*	AOZ17029	*Ancylobacter* sp.	97.9
1	Bacteria	Proteobacteria	Alphaproteobacteria	Hyphomicrobiales	Xanthobacteraceae	*Ancylobacter*	WP_152304407	*Ancylobacter* sp. TS-1	93.9
2	Bacteria	Proteobacteria	Alphaproteobacteria	Hyphomicrobiales	Xanthobacteraceae	*Starkeya*	WP_013166106	*Starkeya novella*	85.4 - 91.7
3	Bacteria	Proteobacteria	Alphaproteobacteria	Hyphomicrobiales	Xanthobacteraceae	*Xanthobacter*	ABS69174	*Xanthobacter autotrophicus* Py2	96.0 - 100
1	Bacteria	Proteobacteria	Alphaproteobacteria	Hyphomicrobiales		*Methylobrevis*	ODN69175	*Methylobrevis pamukkalensis*	93.9
1	Bacteria	Proteobacteria	Alphaproteobacteria	Rhodobacterales	Rhodobacteraceae	*Defluviimonas*	KAB2884096	*Defluviimonas* sp.	87.0
3	Bacteria	Proteobacteria	Alphaproteobacteria	Rhodobacterales	Rhodobacteraceae	*Frigidibacter*	WP_161346160	*Frigidibacter albus*	87.2 - 100
1	Bacteria	Proteobacteria	Alphaproteobacteria	Rhodobacterales	Rhodobacteraceae	*Polymorphum*	WP_013653073	*Polymorphum gilvum*	88.2
1	Bacteria	Proteobacteria	Alphaproteobacteria	Rhodobacterales	Rhodobacteraceae	*Rhodobacter*	WP_008027033	*Rhodobacter* sp. SW2	89.4
1	Bacteria	Proteobacteria	Alphaproteobacteria	Rhodobacterales	Rhodobacteraceae	*Rhodobacter*	WP_135448223	*Rhodobacter* sp. YIM 73028	100
1	Bacteria	Proteobacteria	Alphaproteobacteria	Rhodobacterales	Rhodobacteraceae	*Rubrimonas*	WP_093255626	*Rubrimonas cliftonensis*	100
2	Bacteria	Proteobacteria	Alphaproteobacteria	Rhodobacterales	Rhodobacteraceae	*Tabrizicola*	WP_118150103	*Tabrizicola* sp. DJC	95.9 - 100
1	Bacteria	Proteobacteria	Alphaproteobacteria	Rhodospirillales	Acetobacteraceae	*Humitalea*	WP_111396119	*Humitalea rosea*	78.6
1	Bacteria	Proteobacteria	Alphaproteobacteria	Rhodospirillales	Acetobacteraceae	*Roseomonas*	WP_187777148	*Roseomonas ludipueritiae*	88.6
1	Bacteria	Proteobacteria	Alphaproteobacteria	Rhodospirillales	Azospirillaceae	*Azospirillum*	WP_029010077	*Azospirillum halopraeferens*	92.1
1	Bacteria	Proteobacteria	Alphaproteobacteria	Rhodospirillales	Rhodospirillaceae	*Oceanibaculum*	WP_121218191	*Oceanibaculum indicum*	91.3
2	Bacteria	Proteobacteria	Betaproteobacteria	Burkholderiales	Alcaligenaceae	*Alcaligenes*	AGG56549	*Alcaligenes faecalis*	84.2
1	Bacteria	Proteobacteria	Betaproteobacteria	Burkholderiales	Alcaligenaceae	*Pusillimonas*	WP_129968241	*Pusillimonas soli*	100
5	Bacteria	Proteobacteria	Betaproteobacteria	Burkholderiales	Comamonadaceae	*Acidovorax*	ABY19324	*Acidovorax* sp.	93.8 - 100
1	Bacteria	Proteobacteria	Betaproteobacteria	Burkholderiales	Comamonadaceae	*Curvibacter*	OGP03110	*Curvibacter* sp. GWA2_64_110	100
3	Bacteria	Proteobacteria	Betaproteobacteria	Burkholderiales	Comamonadaceae	*Hydrogenophaga*	BAK39657	*Hydrogenophaga defluvii*	93.8 - 94.0
1	Bacteria	Proteobacteria	Betaproteobacteria	Burkholderiales	Comamonadaceae	*Hydrogenophaga*	MBE0590472	*Hydrogenophaga* sp.	100
4	Bacteria	Proteobacteria	Betaproteobacteria	Burkholderiales	Comamonadaceae	*Limnohabitans*	WP_108365790	*Limnohabitans* sp. Bal53	96.0 - 100
1	Bacteria	Proteobacteria	Betaproteobacteria	Burkholderiales	Comamonadaceae	*Ottowia*	WP_106702863	*Ottowia oryzae*	87.8
3	Bacteria	Proteobacteria	Betaproteobacteria	Burkholderiales	Comamonadaceae	*Ramlibacter*	MBA2672377	*Ramlibacter* sp.	93.0 - 98.0
2	Bacteria	Proteobacteria	Betaproteobacteria	Burkholderiales	Comamonadaceae	*Rhodoferax*	AVY91881	*Rhodoferax* sp.	85.4 - 89.6
1	Bacteria	Proteobacteria	Betaproteobacteria	Burkholderiales	Comamonadaceae	*Rhodoferax*	WP_011465357	*Rhodoferax ferrireducens*	95.8
1	Bacteria	Proteobacteria	Betaproteobacteria	Burkholderiales	Comamonadaceae	*Verminephrobacter*	WP_029554582	*Verminephrobacter aporrectodeae*	91.7
1	Bacteria	Proteobacteria	Betaproteobacteria	Burkholderiales		*Rubrivivax*	WP_128199749	*Rubrivivax albus*	100
2	Bacteria	Proteobacteria	Betaproteobacteria	Nitrosomonadales	Thiobacillaceae	*Thiobacillus*	MBN8758814	*Thiobacillus* sp.	95.1 - 100
2	Bacteria	Proteobacteria	Epsilonproteobacteria	Campylobacterales	Campylobacteraceae	*Sulfurospirillum*	WP_069479145	*Sulfurospirillum halorespirans*	95.8 - 100
1	Bacteria	Proteobacteria	Gammaproteobacteria	Chromatiales	Chromatiaceae	*Thiocapsa*	WP_007194578	*Thiocapsa marina*	100
1	Bacteria	Proteobacteria	Gammaproteobacteria	Pseudomonadales	Pseudomonadaceae	*Pseudomonas*	WP_090181953	*Pseudomonas arsenicoxydans*	96.0
1	Bacteria	Proteobacteria	Gammaproteobacteria	Pseudomonadales	Pseudomonadaceae	*Pseudomonas*	WP_138966343	*Pseudomonas* sp. MPC6	92.1

Table S7: Detected *arrA* in the peat metagenome. Sequences of *arrA* were detected by searching the metagenome against a custom database containing reference sequences and *arrA* obtained in an earlier amplicon-based study from the same site using Blast+. Only matches with a minimum length of 100 bp were taken into account.

No. of matching sequences	Metagenome matches								
	NCBI Taxonomy						Next related sequence		
	Kingdom	Phylum	Class	Order	Family	Genus	Accession no.	Name	% similarity
1	Bacteria	Proteobacteria	Betaproteobacteria	Burkholderiales		*Rubrivivax*	MBL8307377	*Rubrivivax* sp.	97.3
3	Bacteria	Proteobacteria	Betaproteobacteria	Burkholderiales		*Rubrivivax*	MBL8313874	*Rubrivivax* sp.	90.7 - 97.4
2	Bacteria	Proteobacteria	Betaproteobacteria	Burkholderiales		*Rubrivivax*	MBL8323815	*Rubrivivax* sp.	89.8 - 97.0
1	Bacteria	Proteobacteria	Betaproteobacteria	Burkholderiales		*Rubrivivax*	MBL8361870	*Rubrivivax* sp.	98
5	Bacteria	Proteobacteria	Betaproteobacteria	Nitrosomonadales	Sterolibacteriaceae	*Sulfuritalea*	WP_041096744	*Sulfuritalea hydrogenivorans*	81.3 - 97.3
1	Bacteria	Proteobacteria	Gammaproteobacteria	Aeromonadales	Aeromonadaceae	*Aeromonas*	MK163748	*Aeromonas* sp. MrichA-1	100
1	Bacteria	Proteobacteria	Deltaproteobacteria	Desulfobacterales	Desulfobacteraceae	*Desulfatitalea*	KJS28921	*Desulfatitalea* sp.	97.4
2	Bacteria	Proteobacteria	Deltaproteobacteria	Desulfuromonadales	Geobacteraceae	*Geobacter*	ATZ76754	*Geobacter uraniireducens*	89.8
4	Bacteria	Proteobacteria	Deltaproteobacteria	Desulfuromonadales	Geobacteraceae	*Geobacter*	AEK98606	*Geobacter uraniireducens*	88.9 - 94.7
6	Bacteria	Proteobacteria	Deltaproteobacteria	Desulfuromonadales	Geobacteraceae	*Geobacter*	AEK98608	*Geobacter uraniireducens*	81.3 - 87.8
1	Bacteria	Proteobacteria	Deltaproteobacteria	Desulfuromonadales	Geobacteraceae	*Geobacter*	WP_011937411	*Geobacter uraniireducens*	94.9
1	Bacteria	Proteobacteria	Deltaproteobacteria	Desulfuromonadales	Geobacteraceae	*Geobacter*	WP_012469220	*Geobacter lovleyi*	75.8
2	Bacteria	Proteobacteria	Deltaproteobacteria	Desulfuromonadales	Geobacteraceae	*Geobacter*	WP_041970871	*Geobacter* sp. OR-1	89.4 - 89.7
1	Bacteria	Proteobacteria	Epsilonproteobacteria	Campylobacterales	Campylobacteraceae	*Sulfurospirillum*	MW433631	*Sulfurospirillum multivorans*	98
